# Oral Nano-Curcumin in a Model of Chronic Gulf War Illness Alleviates Brain Dysfunction with Modulation of Oxidative Stress, Mitochondrial Function, Neuroinflammation, Neurogenesis, and Gene Expression

**DOI:** 10.14336/AD.2021.0829

**Published:** 2022-04-01

**Authors:** Sahithi Attaluri, Meenakshi Arora, Leelavathi N Madhu, Maheedhar Kodali, Bing Shuai, Laila Melissari, Raghavendra Upadhya, Xiaolan Rao, Adrian Bates, Eeshika Mitra, Keyhan R Ghahfarouki, M. N. V Ravikumar, Ashok K Shetty

**Affiliations:** ^1^Institute for Regenerative Medicine, Department of Molecular and Cellular Medicine, Texas A&M University College of Medicine, College Station, Texas, USA.; ^2^Department of Pharmaceutical Sciences, Irma Lerma Rangel College of Pharmacy, Texas A&M University, College Station, Texas, USA

**Keywords:** cognitive and mood function, hippocampal neurogenesis, inflammasomes, nanoparticles, mitochondria, neuroinflammation, oxidative stress

## Abstract

Unrelenting cognitive and mood impairments concomitant with incessant oxidative stress and neuroinflammation are among the significant symptoms of chronic Gulf War Illness (GWI). Curcumin (CUR), an antiinflammatory compound, has shown promise to alleviate brain dysfunction in a model of GWI following intraperitoneal administrations at a high dose. However, low bioavailability after oral treatment has hampered its clinical translation. Therefore, this study investigated the efficacy of low-dose, intermittent, oral polymer nanoparticle encapsulated CUR (nCUR) for improving brain function in a rat model of chronic GWI. Intermittent administration of 10 or 20 mg/Kg nCUR for 8 weeks in the early phase of GWI improved brain function and reduced oxidative stress (OS) and neuroinflammation. We next examined the efficacy of 12-weeks of intermittent nCUR at 10 mg/Kg in GWI animals, with treatment commencing 8 months after exposure to GWI-related chemicals and stress, mimicking treatment for the persistent cognitive and mood dysfunction displayed by veterans with GWI. GWI rats receiving nCUR exhibited better cognitive and mood function associated with improved mitochondrial function and diminished neuroinflammation in the hippocampus. Improved mitochondrial function was evident from normalized expression of OS markers, antioxidants, and mitochondrial electron transport genes, and complex proteins. Lessened neuroinflammation was noticeable from reductions in astrocyte hypertrophy, NF-kB, activated microglia with NLRP3 inflammasomes, and multiple proinflammatory cytokines. Moreover, nCUR treated animals displayed enhanced neurogenesis with a normalized expression of synaptophysin puncta, and multiple genes linked to cognitive dysfunction. Thus, low-dose, intermittent, oral nCUR therapy has promise for improving brain function in veterans with GWI.

Gulf War Illness (GWI), a chronic multisymptom illness, afflicts ~30% of personnel served in the First GW [1-3]. The GWI-related central nervous system (CNS) symptoms include impaired cognitive and mood function, chronic fatigue, musculoskeletal pain, or other neurological conditions [4-5]. Epidemiological studies have suggested that deployment-related experiences such as the consumption of nerve gas prophylactic drug pyridostigmine bromide (PB), and exposure to insect repellants, insecticides and different types of pesticides employed to stay away from insects and rodents, and war-related stress, caused GWI [4,6]. Exposure to sarin, mustard gas, vaccines, depleted uranium, combustion products, and fuels from burning oil wells were likely the other causes of GWI in veterans [7]. GWI in most veterans is believed to be a consequence of PB's interaction with insecticides and pesticides, or the interaction of several chemicals with the war-related stress [3, 8, 9]. Indeed, animal model studies have shown that 10-28 days exposure to PB and DEET (an insect repellant), PB and permethrin (PER, an insecticide), or PB, DEET, and PER with or without stress results in a GWI phenotype typified by cognitive and mood impairments, neuroinflammation and mitochondrial dysfunction and altered microbiota [10-19]. Similar impairments in cognition and mood and/or neuroinflammation have been seen in other GWI models that employed exposures to sarin surrogate diisopropylfluorophosphate (DFP) with or without a stress component [20-22].

Recent reports from our laboratory have shown that exposure to PB, DEET, and PER with 15 minutes of restraint stress for 28 days is sufficient to induce persistent GWI symptoms. The GWI phenotype observed at 10-12 months post-exposure is characterized by chronic cognitive and mood impairments associated with incessantly raised levels of oxidative stress (OS) markers, inflammatory mediators, reactive astrocytes, activated microglia, and waned hippocampal neurogenesis in the brain [23-25]. We hypothesize that unrelenting cognitive and mood impairments in GWI is a consequence of persistently elevated reactive oxygen species (ROS), mitochondrial hypofunction, and chronic neuroinflammation. Hence, drugs capable of regulating ROS, improving mitochondrial function, and repressing the neuroinflammatory cascade would enhance brain function in GWI. Such premise is corroborated by observations that elevated OS and inflammatory conditions impair hippocampal neurogenesis and cognitive and mood function [26-27], promote mitochondrial hypofunction and synapse loss [28], and alter the expression of genes promoting synaptic plasticity and cognition [29-30].

In this study, we rigorously examined the efficacy of curcumin nanoparticles (i.e., encapsulation of CUR as biodegradable nanosystems, nCUR) for improving cognitive and mood function in a rat model of chronic GWI that involved exposure to PB, DEET, PER and 15 minutes of restraint stress for 28 days [24]. The choice of nCUR is based on our previous finding that intraperitoneal administration of curcumin (CUR) at a higher dose (30 mg/Kg) for 4 weeks commencing immediately after the exposure to GWI chemicals and stress maintained better cognitive function through antioxidant and antiinflammatory activity [15]. The efficacy of CUR has also been established in animal models of several human diseases. These include its ability to enhance hippocampal neurogenesis in the adult brain, improve cognitive function in animal models of Alzheimer’s disease, and ion-radiation via enhancement of canonical Wnt/beta-catenin and/or Nrf-2 antioxidant signaling pathways, and attenuate glutamate neurotoxicity via suppression of inflammasomes [31-35]. Nonetheless, CUR is yet to be approved as a therapeutic agent due to its poor bioavailability in humans following oral administration. Low bioavailability is due to poor absorption and stability at physiological pH, rapid metabolism, and rapid systemic elimination [36-37]. nCUR treatment, on the other hand, has translational potential due to its higher bioavailability than CUR after oral administration [38]. Such increased bioavailability facilitates lower dose and reduced frequency of administration and reduces any potential toxic effects associated with long-term CUR treatment.

First, we tested the efficacy of two different doses of nCUR treatment (10 or 20 mg/Kg) in GWI animals for 8 weeks, starting early (i.e., 2 months) after the exposure. Since both doses moderated several OS and neuroinflammatory markers and improved brain function, we next examined the efficacy of 12-weeks of nCUR treatment at 10 mg/Kg in GWI animals, with treatment commencing 8 months post-exposure. Our results demonstrated that low dose nCUR treatment (10 mg/Kg, 3 times/week) was sufficient for significantly improving cognitive and mood function in rats with chronic GWI. Moreover, such pro-cognitive effects of nCUR were associated with normalization of OS and mitochondrial function markers, repression of inflammatory mediators including NF-kB, NLR family pyrin domain containing 3 (NLRP3) inflammasomes, improved neurogenesis, reduced synapse loss, and normalized expression of genes that promote cognitive function.

## MATERIALS AND METHODS

### Animals

**Figure 1.**
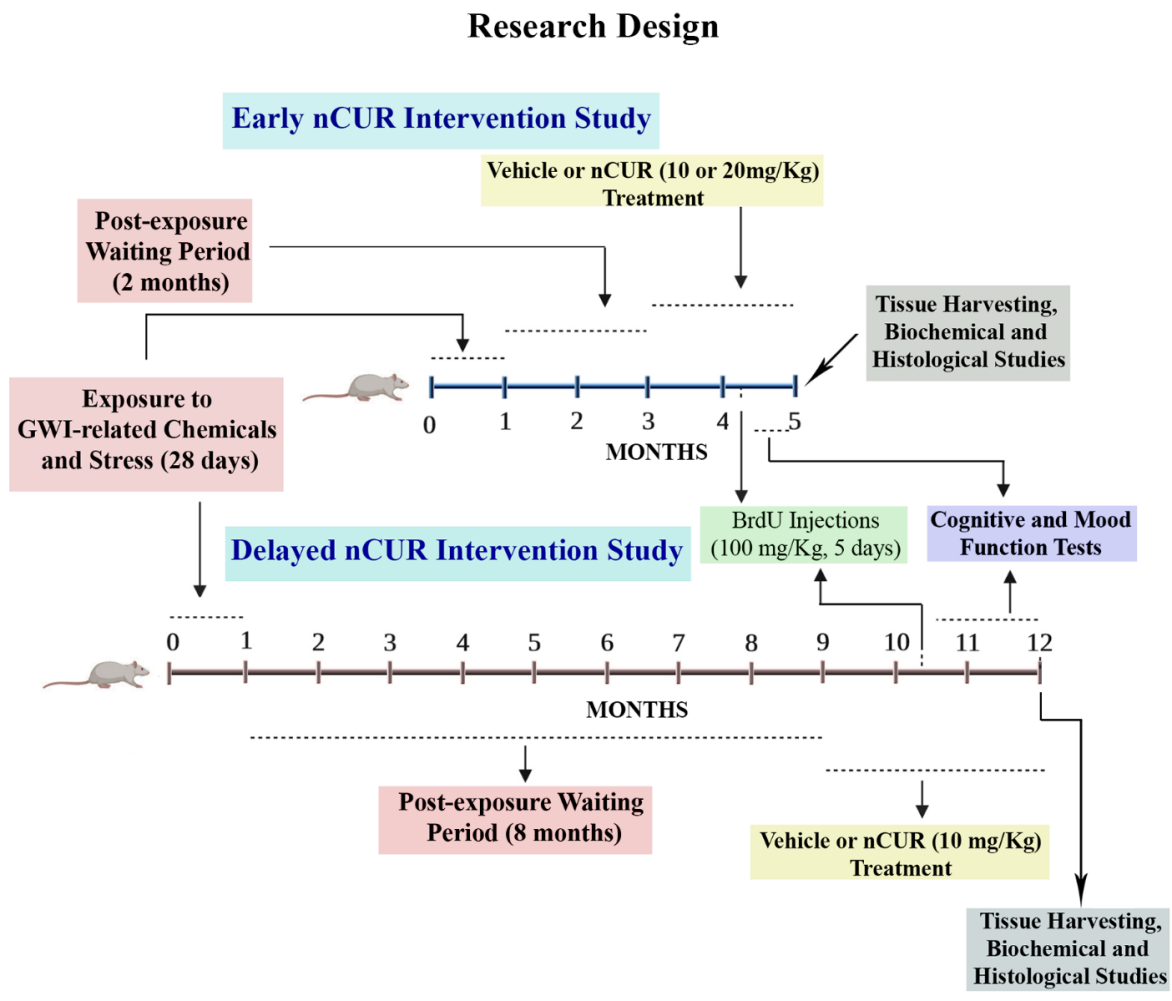
The figure depicts an overview of the experimental design. The top half of the figure shows timelines of the early intervention study with curcumin nanoparticles (nCUR) and brain tissue analyses. Animals were treated with vehicle or nCUR (at 10 mg/20 mg/Kg) for two months, commencing two months after the exposure to Gulf War Illness (GWI)-related chemicals and stress. The behavioral tests were performed in the last three weeks of the vehicle or nCUR treatment, and brain tissues were harvested for biochemical and histological studies. The bottom half of the figure illustrates the timelines of the delayed nCUR intervention study. Animals were treated with vehicle or nCUR (at 10 mg/Kg) for three months, commencing eight months after the exposure to GWI-related chemicals and stress. The behavioral tests were performed in the last six weeks of the vehicle or nCUR treatment, and brain tissues were harvested for biochemical and histological studies.

Approximately two-month-old male Sprague Dawley rats (n=111) were purchased from Harlan (Indianapolis, IN) and housed in the vivarium with *ad libitum* access to food and water. After two weeks of acclimatization, the animals were assigned to either the naïve control group (n=34) or the GWI group (n=77). All animal studies were approved by the institutional animal care and use committee of the Texas A&M Health Science Center College of Medicine. The Figure 1 illustrates the overview of the experimental design and timelines of different experiments performed in the study.

### Exposure of animals to GWIR-chemicals and restraint stress

The animals allotted to the GWI group (n=77) were subjected daily to DEET (200 µl, 60 mg/Kg, dermal; Chem Service Inc, West Chester, PA), PER (200 µl, 0.2 mg/Kg, dermal; Chem Service Inc), and PB (0.5 ml, 2 mg/Kg, oral; Sigma, St. Louis, MO) exposure, and 15 minutes of restraint stress for 28 days, as described in our previous studies [23-25]. Our previous studies have shown that mild or moderate stress alone (5 or 15 minutes of restraint stress) has no adverse effects on brain function [39, and unpublished data], but when combined with GWI-related chemicals, even mild stress exacerbated the effects of chemicals [16]. Age-matched naïve control animals (n=34) did not receive any of the exposures. The chosen chemicals and exposure routes in this study are consistent with epidemiological studies suggesting that GWI in a substantial proportion of GW veterans is an upshot of PB intake and significant exposure to chemicals including DEET and PER, and interaction of these chemicals with stress [3, 8]. Furthermore, the study employed reasonably lower doses of PB, DEET, and PER using routes that mimicked the exposure most veterans underwent during the GW (i.e., PB orally; and PER and DEET dermally). Also, as per the Research Advisory Committee (RAC) report on GWI, the overall incidence of GWI is higher in veterans who were exposed to higher amounts of these chemicals during the GW [8]. Although it is challenging to match the doses of chemicals and the expanse of stress in an animal prototype precisely to those veterans underwent during the GW, the chosen doses did produce the phenotype of GWI exhibiting unremitting cognitive and mood dysfunction, neuroinflammation, and systemic inflammation with no mortality during or immediately after the exposure.

### Preparation and characterization of nCUR

The curcumin nanoparticles (nCUR) employed in this study were formulated by the emulsion-diffusion-evaporation method, as detailed in previous reports [38, 40-43]. Briefly, 75 mg of CUR (Fisher Scientific, Hampton, NH) dissolved in 5 ml of ethyl acetate was added to a polylactide-co-glycolide (PLGA, 500mg) solution in 20 ml of ethyl acetate, followed by stirring (1,000 rpm) at room temperature for one hour. Next, this organic phase was added dropwise to 50 ml of 1% (w/v) polyvinyl alcohol (PVA) in water to prepare an oil-in-water emulsion. The emulsion was then subjected to homogenization at 15,000 rpm for 30 minutes, added to excess water (250 ml), and stirred overnight to evaporate the organic solvent. The suspension was centrifuged at 15000g for 30 min at 40C. The pellet was resuspended in 20 mL of 5% sucrose solution. Ten ml aliquots of this suspension were added to 20 ml vials and freeze-dried at -55°C for 54 h, followed by heating at 20°C for 20 h under vacuum (0.008 mbar) [Labconco FreeZone Triad]. Upon removal from the freeze drier, the vials were crimp sealed and stored at 4°C until further use. Three vials of nCUR from each batch were picked to measure the particle size using a particle size analyzer (Malvern, UK), and the CUR content was quantified using the prior developed HPLC method [40,42,44]. The particles were 250 nm in size and contained about 0.15 mg of CUR per milligram PLGA.

### nCUR Treatment to GWI animals

We first investigated the efficacy of two different doses of oral nCUR treatment (10 or 20 mg/Kg, three times/week with each treatment separated by 1-2 days) in GWI animals for 8 weeks, starting 2 months after the exposure to chemicals and stress. The required amount of nCUR suspension was freshly prepared in sterile water on the day of administration via oral gavage. The early intervention study comprised the following animal groups: (1) GWI-vehicle (GWI-VEH) group receiving empty nanoparticles (n=16); (2) GWI-nCUR10 group receiving 10 mg/kg nCUR (n=16); (3) GWI-nCUR20 group receiving 20 mg/kg nCUR (n=16); and (4) an age-matched naïve control group (n=18). Animals received intraperitoneal injections of 5’-bromodeoxyuridine (BrdU, 100 mg/Kg) for 5 days in the 5^th^ week of the treatment for measuring hippocampal neurogenesis. Both doses (10 or 20 mg/Kg) of oral nCUR in the early intervention study were equally effective in moderating several OS and neuroinflammatory markers and improving cognitive and mood function. Therefore, in the delayed intervention study, we chose the lower dose (10 mg/Kg, three times/week, oral) for examining the efficacy of 12-weeks of nCUR treatment in animals with chronic GWI, in which nCUR treatment commenced 8 months after the exposure to chemicals and stress. An extended waiting period of 8 months after the exposure was chosen to commence nCUR administration to simulate the treatment of chronic GWI in veterans. The eight-month time point after exposure in rats is equivalent to ~24 years of survival after exposure in humans [45]. The delayed intervention study included the following animal groups: (1) GWI-vehicle (GWI-VEH) group receiving empty nanoparticles, (n=15); and (2) GWI-nCUR group receiving 10 mg/kg nCUR (n=14); (3) an age-matched naïve control group (n=16). Animals received intraperitoneal injections of BrdU (100 mg/Kg) for 5 days in the 7^th^ week of the treatment for assessing hippocampal neurogenesis. Age-matched naïve control animals did not receive nCUR or vehicle treatment but received comparable BrdU injections and behavioral testing at time-points matching the GWI groups.

### Time-points of behavioral tests for assessing cognitive and mood function

A series of behavioral tests were employed to assess cognitive and mood function in all animal groups belonging to early and delayed intervention studies. The tests comprised an object location test (OLT), a novel object recognition test (NORT), and a sucrose preference test (SPT). The behavioral tests were done during the last 4 weeks of nCUR/VEH treatment in early and delayed intervention studies. Such time-points were equivalent to analysis at ~3 months post-exposure to chemicals and stress in the early intervention study and ~10 months post-exposure to chemicals and stress in the delayed intervention study.

### Hippocampus-dependent cognitive function analysis via an OLT

**Figure 2.**
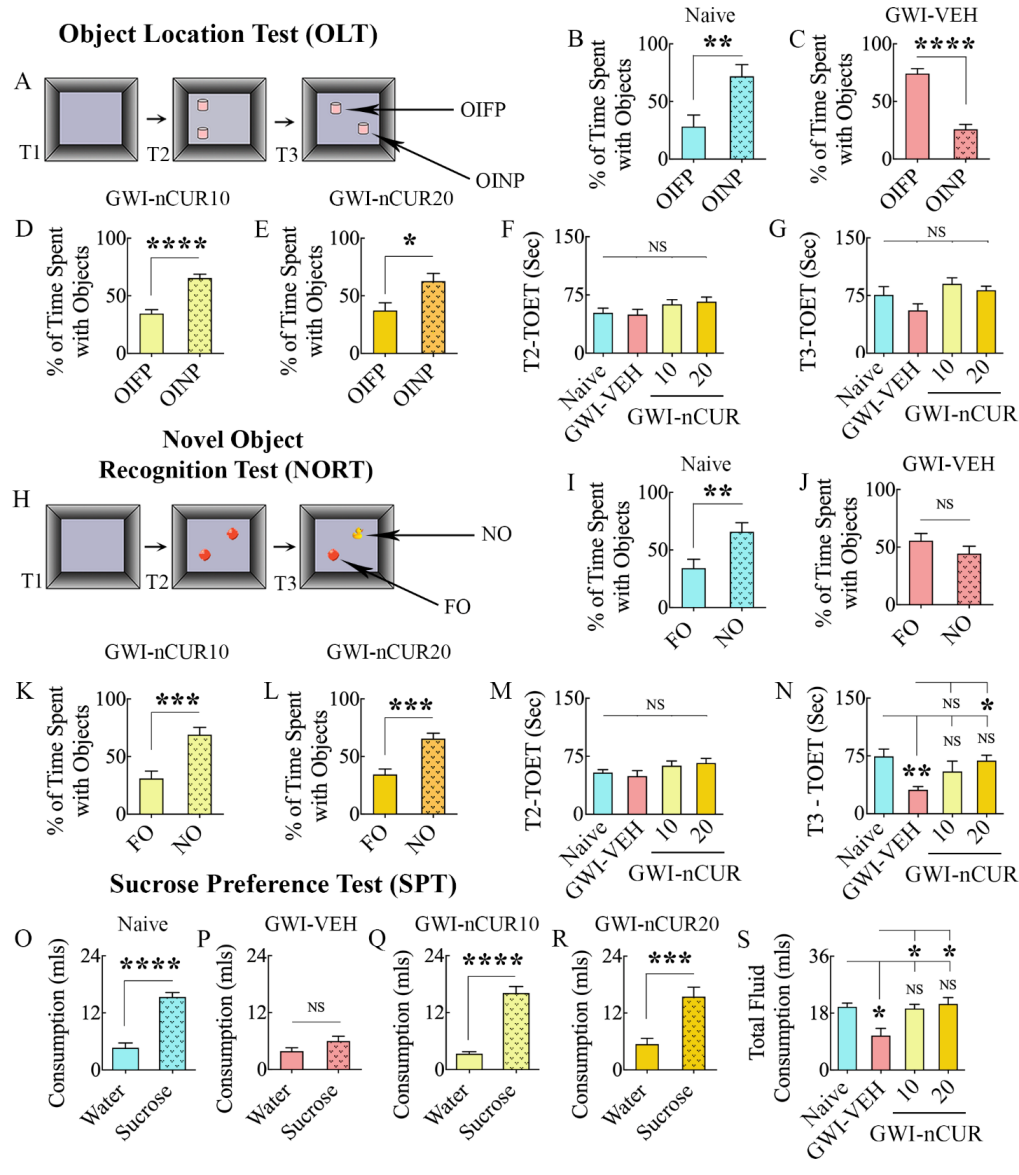
Eight weeks of nCUR treatment, commencing 2 months after exposure to GWI-related chemicals and stress, improved cognitive and mood function. The bar charts in the top two rows show the results of an object location test (OLT). Cartoon (A) shows the various phases involved in OLT whereas, the bar charts in (B-E) show the performance of animals belonging to naïve and GWI rats receiving vehicle (GWI-VEH) or different doses of nCUR (GWI-nCUR10, GWI-nCUR20). The animals in the naïve group (B) preferred the object in a novel place (OINP) over the object in a familiar place (OIFP), implying their ability to discern minor changes in the environment. Animals in the GWI-VEH group (C) were impaired, as they preferred the OIFP over the OINP, whereas animals in nCUR10 (D) and nCUR20 (E) groups behaved akin to the naive control group, implying an improved cognitive function. The bar charts (F-G) compare the total object exploration times (TOETs) in trial-2 (T2) and T3. The bar charts in rows 3-4 show the results of a novel object recognition test (NORT). Cartoon H shows the various phases involved in NORT, whereas the bar charts in (I-L) show the performance of animals belonging to different groups. The animals in the naïve group (I) preferred the novel object (NO) over the familiar object (FO), suggesting their proficiency for object recognition memory. Animals in the GWI-VEH group (J) were impaired, as they did not prefer NO, whereas animals in nCUR10 (K) and nCUR20 (L) groups behaved similarly to the naive control group, implying an improved recognition memory function. The bar charts (M-N) compare the TOETs in T2 and T3. The bar charts in the last row (O-S) show the results of a sucrose preference test. Animals in the naïve group (O) preferred sucrose-containing water over the standard water. Animals in the GWI-VEH group (P) exhibited anhedonia as they did not prefer the sucrose-containing water, whereas animals in GWI-nCUR10 (R) and nCUR20 (S) groups preferred to drink the sucrose-containing water, implying no anhedonia. The bar chart in S compares the total fluid consumption across groups. *, p<0.05, **, p<0.01, ***, p<0.001, and ****, p<0.0001; NS, not significant.

The animals were investigated with an OLT to ascertain their proficiency for discerning minor changes in the environment, as described in our previous reports (n=8-9/group in the early intervention study and n=12-16/group in the delayed intervention study) [14,15,24]. In brief, each animal was given three trials (T1, habituation trial; T2, sample trial; T3, test trial). Each trial lasted for 5 minutes with an inter-trial interval (ITI) of 60 minutes (Fig. 2). Each animal explored the empty open field apparatus in T1, and the two identical objects placed on opposed sides of the box in T2. In T3, the animal explored the same objects from T2, but one of the objects moved to a novel location in the open field. The movement of the rat in T2 and T3 were video tracked using Anymaze software. Because the robustness of OLT needs exploring both objects and their settings in T2 and significant total object exploration time (TOET) in T3 (Fig. 2), we established inclusion criteria for data analysis. Only animals that explored objects ≥16 seconds in T2 and ≥8 seconds in T3 were included. In every group, the extent of cognitive function was confirmed by comparing the percentages of time spent exploring the object in the familiar place (OIFP) with the percentages of time spent exploring the object in the novel place (OINP) in T3. Furthermore, data such as the TOETs, the total distance traveled, and movement velocity were computed for both T2 and T3 and compared across groups.

### Recognition memory evaluation through a NORT

The animals were probed with a NORT to assess their recognition memory competence, as detailed in our previous reports (n=8-10/group in the early intervention study and n=13-16/group in the delayed intervention study) [14, 23]. Briefly, the test comprised three trials (T1-T3), each of which lasting 5 minutes with an ITI of 30 minutes (Fig. 2). As in OLT, the first two trials encompassed the exploration of an empty open field apparatus (T1) and exploration of two indistinguishable objects positioned on opposing sides of the open field apparatus (T2). In T3, the animal explored one of the objects from T2 remaining in the same location (familiar object, FO), but a novel object (NO) replaced the second object. Animals' movement in T2 and T3 were video tracked using Anymaze software. As in OLT, to maintain the test's validity, only animals that explored objects ≥16 seconds in T2 and ≥8 seconds in T3 were included for data analysis. In each group, the extent of recognition memory function was verified by comparing the percentages of time spent exploring the NO vis-à-vis the FO in T3. Besides, the TOETs, the total distance traveled, and the velocity of movement were compared across groups for both T2 and T3.

### Assessment of anhedonia through a SPT

We employed a SPT to assess anhedonia, which comprised monitoring animals for four consecutive days as detailed in our previous report (n=9/group in the early intervention study and n=11-15/group in the delayed intervention study) [24]. Briefly, on day 1, the animals were housed individually and provided with ad libitum food and two identical bottles containing 1% sucrose solution. On day 2, one of the sucrose-containing bottles was replaced with a bottle containing standard water. On day 3, animals were deprived of both food and water for 22 hours. On day 4, animals were given access to two bottles, one with 1% sucrose solution and the other with standard water, for two hours. The amount of sucrose-containing water and standard water consumption was measured during the two-hour testing phase on day 4, and the animals were placed back in their home cages and provided with ad libitum food and water. A decreased preference for sucrose-containing water over the standard water implies anhedonia in this test. In each group, anhedonia manifestation was gauged by comparing the volume of sweet water consumption with regular water consumption.

### Brain tissue harvesting for molecular and immunohistochemical studies

Subgroups of animals from early and delayed intervention studies (n=7-10/group) were deeply anesthetized and euthanized by decapitation [17]. The fresh brain was quickly removed from each of these animals and snap-frozen using dry ice and stored in a -80°C freezer until further analysis through biochemical or molecular biological assays. Additional subgroups of animals from both early and delayed intervention studies (n=7-8/group) were deeply anesthetized and perfused with 4% paraformaldehyde, as described in our previous reports [46-48]. These fixed brain tissues were used for various immunohistochemical and immunofluorescence studies. The time-points of tissue analysis performed were equivalent to ~4 months after exposure to chemicals and stress in the early intervention study and ~11 months after exposure to chemicals and stress in the delayed intervention study.

### Preparation of hippocampal tissue samples for biochemical and gene expression studies

Both hippocampi were first micro-dissected from each of the freshly harvested brains in all groups. The hippocampus from one side in each animal was used for preparing the hippocampal tissue lysate using methods detailed in our previous studies [17, 23-25, 49]. The hippocampal tissue lysate was used to measure OS markers, antioxidants, mitochondrial complex proteins, proinflammatory cytokines, and other proteins (n=5-6/group). The hippocampus from the other side in each animal was processed to analyze the expression of genes encoding proteins relevant to regulating mitochondrial respiratory chain and cognitive function, using a qRT-PCR array (n=5-7/group).

### Measurement of OS markers, antioxidants, and NRF2

We measured OS markers from hippocampal tissue lysates using commercially available kits, as described in our previous reports (n=5-8/group) [17, 24-25]. Manufacturer’s instructions were followed for measuring malondialdehyde (MDA, Cayman chemicals, Arbor, MI), protein carbonyls (PC, Cayman), superoxide dismutase (SOD, Cayman), and nuclear factor [erythroid-derived 2]-like 2 (Nrf-2, Signosis, Santa Clara, CA).

### Measurement of mitochondrial complex proteins, NF-kB, NLRP3, caspase-1, and proinflammatory cytokines and chemokines

We employed well-standardized, commercially available kits for measuring mitochondrial complex proteins (complex I, II, III, and IV), NF-kB, NLRP3, caspase-1, and other proinflammatory cytokines and chemokines (n=5-8/group). The kits were purchased from Abcam (Cambridge, MA) for measuring mitochondrial complexes I and IV, and from MyBioSource (San Diego, CA) for quantifying mitochondrial complex II and III. We estimated the relative levels of 16 cytokines/chemokines using a quick and sensitive rat cytokine array kit from Signosis (Santa Clara, CA). We also employed individual ELISA kits to measure the concentration of NF-kB (Aviva Systems Biology, San Diego, CA, detection range: 78-5000pg/ml), NLRP3 (Aviva Systems Biology, San Diego, CA, detection range: 0.312-20 ng/ml), caspase-1 (R&D Systems, Minneapolis, MN), tumor necrosis factor-alpha (TNF-α, Signosis, detection range: 0.05-4000 pg/ml), interleukin-1 beta (IL-1β, Signosis, detection range: 0.05-4000 pg/ml), and IL-18 (R&D Systems, detection range: 62.5-4000pg/ml). We followed the manufacturer’s instructions and the methods described in our previous reports [17, 23-25].

### Measurement of genes related to mitochondrial respiratory chain and cognitive function via real-time polymerase chain reaction (qRT-PCR)

We analyzed the expression of many genes regulating mitochondrial respiratory chain and cognitive function in the hippocampus, using specific primers purchased from Integrated DNA Technologies IDT (Coralville, IW) (n=5-8/group). The methods for the total RNA extraction using RNeasy kit (Qiagen, Valencia, CA), the conversion of total RNA into cDNA using the RT2 First Strand Kit (Qiagen) are available in our previous reports [17, 24, 50]. The measured genes encoding proteins relevant to the mitochondrial electron transport chain and oxidative phosphorylation include the NADH: ubiquinone oxidoreductase subunit S6 and S7 (*Ndufs6 and Ndufs7* - complex I), succinate dehydrogenase A and B (*SdhA and SdhB* - complex II), BCS1 homolog, ubiquinol-cytochrome C reductase complex chaperone (*Bcs1l* - Complex III) *and* cytochrome C1 (*Cyc1* - complex III)*;* Cytochrome c oxidase subunit 6a (*Cox6a* - complex IV) and cytochrome C oxidase subunit 4i2 (*Cox4i2* - complex IV), ATPase H+ transporting accessory protein 1 (*Atp6ap1* - complex V), and Solute Carrier Family 25 Member 10 (*Slc25a10* - mitochondrial membrane transporter). The measured genes related to cognitive function comprised brain-derived neurotrophic factor (*Bdnf*), fibroblast growth factor (*Fgf*), glutamate receptor, ionotropic, N-methyl D-aspartate 2A (*Glun2a*), *Glun2b*, mitogen-activated protein kinase 1 (*Mapk1*), *Mapk3*, Metabotropic glutamate receptor 5 (*Mglu5*), NMDA receptor-regulated 2 (*Narg2*), glyoxylate reductase 1 homolog (*N-pac*), cAMP-specific 3',5'-cyclic phosphodiesterase 4B (*Pde4b*), Ca^2+^/calmodulin-dependent protein kinase II (*CamkII*). The reactions were done as per the manufacturer’s protocol using a CFX96 Real-Time System (Bio-Rad, Hercules, CA). The PCR amplification and melt curve analysis were performed, and Ct values (threshold cycle) were obtained [15, 17, 51]. For calculating delta Ct values, the Ct values were exported to an Excel spreadsheet and analyzed using web-based Qiagen PCR array data analysis software. Hypoxanthine phosphoribosyltransferase *(Hprt)* and Glyceraldehyde 3-phosphate dehydrogenase *(Gapdh)* were used as housekeeping genes in the mitochondrial gene expression study, and *Hprt* was used as the housekeeping gene in the cognitive function gene expression study. The 2^delta Ct values for each gene were compared across different groups.

### Immunohistochemistry

We performed immunohistochemistry for visualizing glial fibrillary acidic protein (GFAP) positive astrocytes, BrdU+ newly born cells, and doublecortin (DCX) positive newly born neurons in the hippocampus. The methods employed for cutting thirty-micrometer thick coronal sections through the entire forebrain, collection, and storage of sections in cryobuffer, and immune-histochemical methods are described in our previous reports [15, 46, 52, 53]. GFAP, BrdU, and DCX immunohistochemistry were done on every 15th or 20th section through the hippocampus's entire septotemporal axis in each animal. The primary antibodies comprised rabbit anti-GFAP (1:2000; Dako, Santa Clara, CA), mouse anti-BrdU (1:500; Abcam), and goat polyclonal anti-DCX (1:300; Santa Cruz, Dallas, TX). The secondary antibodies comprised anti-rabbit, anti-mouse, or anti-goat IgG’s (Vector Laboratories, Burlingame, CA). The avidin-biotin complex reagent and chromogen kits (vector gray or diaminobenzidine) were also purchased from Vector Labs. The sections were fixed on gelatin-coated slides, dehydrated, cleared, and coverslipped.

### Quantification of hypertrophied astrocytes and percentages of microglia expressing ED-1

The hypertrophy of astrocytes in different subfields of the hippocampus was evaluated by measuring the area fractions occupied by GFAP immunoreactive structures using Image J (n=5-6/group). The percentages of activated microglia (i.e., ED-1+ cells) among all microglia (i.e., Ionized calcium binding adaptor molecule 1-positive [IBA-1+] cells) were measured through dual immunofluorescence for IBA-1 and ED-1, as described in our previous report (n=5-6/group) [24-25]. Z-section imaging was done using a Nikon confocal microscope to calculate the percentage of IBA-1+ cells expressing ED-1. These percentages were compared across groups. Goat anti-IBA-1 (1:1000, Abcam), mouse anti-ED-1 (1:1000, Bio-Rad, Hercules, CA), donkey anti-goat IgG tagged with Alexa Flour 488 (1:200, Invitrogen, Grand Island, NY), and donkey anti-mouse IgG tagged with Alexa Flour 594 (1:200, Invitrogen) were used in these studies.

### Measurement of hippocampal neurogenesis

The numbers of BrdU+ newborn cells in the subgranular zone-granule cell layer (SGZ-GCL) of the dentate gyrus were measured via stereology. Such analyses estimated numbers of new cells born in the 5^th^ week of nCUR/VEH treatment (equivalent to ~3 months after exposure to chemicals and stress) and survived for 3 weeks in the early intervention study (n=6/group). In the delayed intervention study, the analysis quantified numbers of new cells born in the 7^th^ week of nCUR/VEH treatment (equivalent to ~9.5 months post-exposure to chemicals and stress) and survived for 6 weeks (n=5-6/group). The status of hippocampal neurogenesis at the time of euthanasia was measured through stereological quantification of newly born, DCX+ neurons in the SGZ-GCL of the dentate gyrus (n=6/group). Such analyses estimated numbers of new neurons born: (i) after 8 weeks of nCUR/VEH treatment (i.e., at ~4 months after exposure to chemicals and stress) in the early intervention study); and (ii) after 12 weeks of nCUR/VEH treatment (i.e., at ~11 months after exposure to chemicals and stress in the delayed intervention study). Stereological counting of BrdU+ and DCX+ cells utilized every 15th section through the entire hippocampus (n=6/group), as described elsewhere [48, 54, 55]. Moreover, percentages of newly born cells (i.e., BrdU+ cells) that differentiated into neurons and net neurogenesis were estimated through BrdU and neuron-specific nuclear antigen (NeuN) dual immunofluorescence and Z-section analyses in a confocal microscope [53]. Mouse anti-NeuN (1:1000, Millipore, Burlington, MA), rat anti-BrdU (1:500, Abcam), donkey anti-mouse IgG tagged with Alexa Flour 488 (1:200, Invitrogen, Grand Island, NY), and donkey anti-rat IgG tagged with Alexa Flour 594 (1:200, Invitrogen) were used in these studies. Two-micrometer thick optical Z-sections were employed to quantify the percentages of BrdU+ cells that expressed NeuN.

### Analysis of the presynaptic and postsynaptic protein density

We visualized presynaptic and postsynaptic puncta through dual immunofluorescence staining of brain tissue sections for synaptophysin (Syn) and postsynaptic density protein-95 (PSD95) and then we performed 0.5-micrometer thick Z-sectioning in a Leica THUNDER 3D imaging system (n=5-6/group). Rabbit anti-Syn (1:500, Synaptic Systems, Goettingen, Germany), goat anti-PSD95 (1:500, Abcam), donkey anti-rabbit IgG tagged Alexa Fluor 488 (1:200, Invitrogen), and donkey anti-goat IgG tagged Alexa Fluor 594 (1:200, Invitrogen) were used in these studies. We employed Image J and blinded analysis to quantify the area fraction of Syn+ and PSD95+ puncta in 303 µm^2^ areas of the dentate gyrus' molecular layer (2 sections/animal) [56].

### Quantification of Inflammasomes

We visualized NLRP3 inflammasomes in microglia, neurons, and astrocytes through triple immune-fluorescence staining of brain tissue sections (n=6/group) for NLRP3, an apoptosis-associated speck-like protein containing a CARD (ASC), and IBA-1, NeuN, or GFAP. Goat anti-NLRP3 (1:500, Millipore), mouse anti-ASC (1:1000, Santa Cruz), rabbit anti-NeuN (1:1000, Millipore), rabbit anti-GFAP (1:1000, Millipore), rabbit anti-iba1 (1:1000 Abcam), donkey anti-goat IgG tagged Alexa Fluor 488 (1:200, Invitrogen), donkey anti-mouse Alexa Fluor 594 (1:200, Invitrogen), and donkey anti-rabbit 405 (1:200, Invitrogen) were employed in these studies. Two-micrometer thick optical Z-sections were employed for blinded quantification of the total number of NLRP3 inflammasomes (i.e., structures positive for both NLRP3 and ASC) in 216 μm^2^ area of the CA3 subfield of the hippocampus (2 sections/animal, n=5-6/group). We also quantified the percentages of IBA-1+ microglia, NeuN+ neurons, and GFAP+ astrocytes containing NLRP3 inflammasomes (2 sections/animal) [25].

### Measurement of Wnt3a and beta-Catenin

We measured Wnt3a and beta-catenin proteins in the hippocampal tissue lysates to understand the possible mechanisms underlying the improved neurogenesis with nCUR treatment in the delayed intervention study (n=5-6/group). We used quantitative sandwich ELISA kits for measuring Wnt3a (Aviva system biology, detection range: 0.312-20ng/ml) and beta-catenin (Aviva system biology, detection range, 0.156-10ng/ml) in the hippocampus by following protocols supplied by the manufacture.

### Statistical analyses

We employed a two-tailed, unpaired Student’s t-test (or Mann-Whitney U-test when standard deviations varied significantly) for comparisons between two datasets. We employed one-way ANOVA with Newman-Keuls multiple comparison post-hoc tests for comparisons involving three or more datasets. We performed the Kruskal Wallis test with Dunn’s post hoc tests when individual groups did not pass the normality test (Shapiro-Wilk test). In all comparisons, p<0.05 was considered a statistically significant value.

## RESULTS

### A. Effects of early nCUR intervention after exposure to GWI-related chemicals and stress

We first ascertained the efficacy of 8 weeks of nCUR treatment at 10 or 20 mg/Kg (3 days/week) in GWI animals, starting two months after the exposure to chemicals and stress.

### Early nCUR intervention improved cognitive function, recognition memory, and mood

We first determined the hippocampus-dependent cognitive function through an OLT, which measured the animal’s proficiency to recognize minor changes in their immediate environment (Fig. 2A). All animals tested (n=8-9/group) met the criteria employed for this task (i.e., exploration of objects ≥16 seconds in T2 and ≥8 seconds in T3). A preference to inspect the OINP over the OIFP in T3 authenticated the competency of naïve animals for this cognitive task (t=3.1, p<0.01, Fig. 2B). Animals belonging to the GWI-VEH group exhibited impaired cognitive function, which was apparent from their exploration of the OIFP for more extended periods than the OINP (t=8.1, p<0.0001, Fig. 2C). In contrast, GWI animals receiving nCUR (10 or 20 mg/Kg) displayed improved cognitive function, as they explored the OINP for longer durations than the OIFP (t=2.7-6.5, p<0.05-0.0001 Fig. 2D-E). The TOETs in T2 and T3 were comparable across the groups (H=5.2-7.2, p>0.05, Fig. 2F-G).

The animals were next tested for recognition memory function using a NORT (Fig. 2H), which depends on the integrity of both the perirhinal cortex and the hippocampus. The test determined the competence for distinguishing the NO from the FO. Again, most animals (7-10/group from 8-10 tested/group) met the yardsticks used for this task (i.e., exploration of objects ≥16 seconds in T2 and ≥8 seconds in T3). Intact recognition memory function in naïve animals was confirmed by their exploration of the NO for a longer duration than the FO (t=2.9, p<0.01, Fig. 2I). Animals in the GWI-VEH group displayed recognition memory impairment, which was visible from their exploration of both NO and FO for comparable periods (t=1.3, p>0.05, Fig. 2J). However, GWI animals receiving nCUR (10 or 20 mg/Kg) exhibited proficiency for recognition memory, as they explored the NO for a longer duration than the FO (t=4.2-4.7, p<0.001, Fig. 2K-L). The TOETs were comparable across the groups in T2 (H=5.2, p>0.05, Fig. 2M). In T3, the TOETs varied between groups, the GWI-VEH group explored less than the naive control group (F=4.5, p<0.01, Fig. 2N).

After cognitive tests, an SPT was employed to investigate anhedonia (n=9/group), a common symptom of depression in which the activities that are gratifying when healthy are no longer pleasurable. Naïve control animals exhibited no anhedonia, which was evident from their preference to drink more sucrose-containing water than regular water (t=7.8, p<0.0001, Fig. 2O). Animals in the GWI-VEH presented anhedonia through drinking nearly equivalent amounts of sucrose-containing water and regular water (t=0.7, p>0.05, Fig. 2P). However, GWI animals receiving nCUR (10 or 20 mg/Kg) showed a behavior similar to naïve control rats. They preferred sucrose-containing water over normal water (t=4.3-8.8, p<0.001-0.0001, Fig. 2Q-R). The overall fluid consumption (i.e., sucrose-containing water plus regular water) varied between groups (H=12.2, p<0.01, Fig. 2S). Compared to the naive control group, the consumption was reduced in the GWI-VEH group (p<0.05) but similar in GWI-nCUR groups (p>0.05, Fig. 2S). Thus, 10-20 mg/Kg doses of nCUR treatment for 8 weeks were sufficient to improve cognitive, memory, and mood function in GWI rats when treatment intervention commenced two months after exposure to GWI related chemicals and stress.

### Early nCUR intervention eased OS, mitochondrial dysfunction, and proinflammatory cytokine levels in the hippocampus of GWI rats

**Figure 3.**
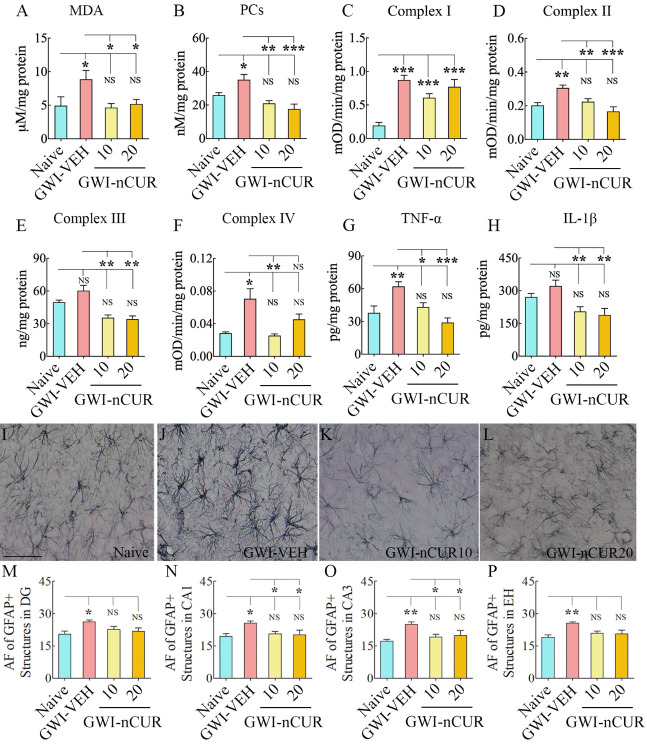
Eight weeks of nCUR treatment, commencing 2 months after exposure to GWI-related chemicals and stress, reduced oxidative stress, mitochondrial dysfunction, and inflammation in the hippocampus. The bar charts in the top two rows compare the concentrations of malondialdehyde (MDA; A), protein carbonyls (PCs; B), mitochondrial complexes I (C), II (D), III (E), and IV (F), tumor necrosis factor-alpha (TNF-α; G), and interleukin-1 beta (IL-1β; H) between naïve rats and GWI rats receiving vehicle (GWI-VEH) or different doses of nCUR (GWI-nCUR10, GWI-nCUR20). Compared to the naïve control group, nCUR treatment at both doses normalized the MDA and PCs (A-B), mitochondrial complexes II and IV (D, F), and TNF-α (G) to naïve control levels. Compared to the GWI-VEH group, the concentration of complex III (E) and IL-1β (H) were also reduced in nCUR groups. (I-L) show examples of GFAP+ astrocytes from the CA3 subfield of naïve control (I), GWI-VEH (J), GWI-nCUR10 (K), and GWI-nCUR20 (L) groups. The bar charts M-P compare the area fraction (AF) of GFAP+ structures in the DG (M), the CA1 subfield (N), the CA3 subfield (O), and the entire hippocampus (P) between different groups. Note that the AF of GFAP+ structures is higher in the GWI-VEH group, but AF’s in all hippocampal regions were normalized to naïve control levels in both nCUR groups. Scale bar, I-L = 100 μm. *, p < 0.05, **, p < 0.01, and ***, p < 0.001; NS, not significant.

To discern the effects of early nCUR intervention on OS and neuroinflammation in the hippocampus of GWI rats, we measured OS markers (MDA and PCs), the mitochondrial complex proteins (complex I, II, III, and IV), and proinflammatory cytokines (TNF-α and IL-1β). Evaluation using ANOVA showed differences between groups for both MDA and PCs (F=3.6-10.1, p<0.05-0.001, Fig. 3A-B). The levels of MDA and PCs were elevated in the GWI-VEH group, in comparison to the naïve control group (p<0.05), but were normalized to control levels in GWI rat groups receiving either 10 or 20 mg/Kg doses of nCUR (p>0.05 versus the naïve control group, and p<0.05-0.001 versus the GWI-VEH group, Fig. 3A-B). Analyses for mitochondrial complex proteins also showed differences between groups (F/H= 8.8-18.4, p<0.01-0.0001, Fig. 3C-F). The animals in the GWI-VEH group displayed the increased activity of mitochondrial complex proteins I, II and IV (p<0.05-0.001, Fig. 3C-D, F), implying hyperactive mitochondria at this stage of GWI (i.e., ~4 months after exposure to chemicals and stress), which is consistent with our previous results [15, 17]. nCUR treatment did not significantly alter the complex I activity (p<0.001 versus the naïve control group, Fig. 3C). However, both doses of nCUR treatment normalized the activity of complex II and IV to naïve control levels (p>0.05, Fig. 3D, F). Thus, early nCUR treatment after exposure to GWI chemicals and stress significantly reduced OS markers and the activity of several mitochondrial complex proteins in the hippocampus, implying a robust antioxidant effect.

Next, we investigated whether nCUR treatment reduced the concentration of proinflammatory cytokines TNF-α and IL-1β. Analysis using ANOVA showed differences between groups for both TNF-α and IL-1β (F=6.5-8.2, p<0.01, Fig. 3G-H). The concentration of TNF-α was higher in the GWI-VEH group, compared to the naïve control group (p<0.01), but was normalized to control levels in GWI rat groups receiving either dose of nCUR (p>0.05 versus the naïve control group, and p<0.05-0.001 versus the GWI-VEH group, Fig. 3G). The concentration of IL-1β showed a similar trend, but the increase in the GWI-VEH group was not significant compared to the naïve control group (p>0.05), Fig. 3H). Nonetheless, the GWI rat groups receiving nCUR displayed reduced IL-1β levels than the GWI-VEH group (p<0.01, Fig. 3H). Thus, early intervention with nCUR after exposure to GWI chemicals and stress maintained a reduced concentration of major proinflammatory cytokines in the hippocampus, implying an anti-inflammatory effect.

### Early nCUR therapy reduced astrocyte hypertrophy and microglial activation in the hippocampus of GWI rats

To further validate the antiinflammatory effects of nCUR, we quantified the extent of astrocyte hypertrophy in the hippocampus. The animals in the GWI-VEH group displayed significant astrocyte hypertrophy in all hippocampal subfields compared to the naïve control group, but animals that received nCUR displayed reduced hypertrophy of astrocytes. Examples of GFAP+ astrocytes in the CA3 subfield of the hippocampus from different groups are illustrated in Fig. 3I-L. Comparison of area fraction of GFAP+ astrocytic elements revealed significant differences between groups for individual subfields and when the hippocampus was taken in its entirety (F/H=3.4-12.8, p<0.05-0.01, Fig. 3M-P). Increased hypertrophy was observed in animals belonging to the GWI-VEH group compared to the naïve control group (p<0.05-0.01, Fig. 3M-P). nCUR treatment at both doses normalized astrocyte elements to naïve control levels, however (p>0.05, Fig. 3M-P). Furthermore, the extent of astrocytic elements in GWI-nCUR groups was significantly reduced compared to the GWI-VEH group for the CA1 and CA3 subfields (p<0.05, Fig. 3N-O). Thus, nCUR treatment at 10-20 mg/Kg doses reversed astrocyte hypertrophy in GWI rats.

**Figure 4.**
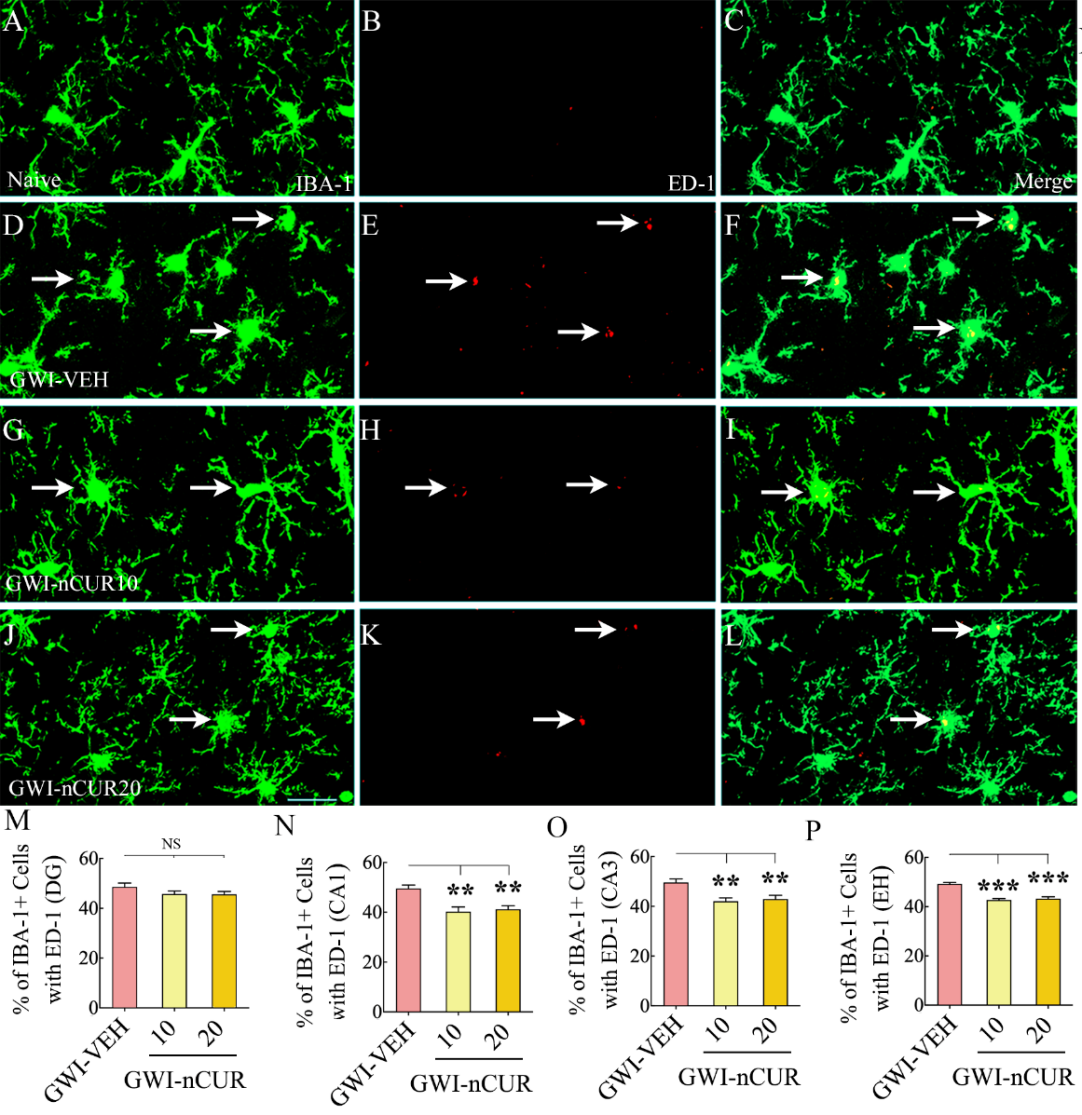
Eight weeks of nCUR treatment, commencing 2 months after exposure to GWI-related chemicals and stress, reduced activated microglia in the hippocampus. Figures A-L illustrate examples of IBA-1+ microglia expressing ED-1 in the hippocampus of naïve (A-C) and GWI rats receiving vehicle (GWI-VEH; D-F) or different doses of nCUR (GWI-nCUR10, G-I; GWI-nCUR20; J-L). Bar charts M-P illustrate the percentages of IBA-1+ microglia expressing ED-1 in the dentate gyrus (DG; M), CA1 subfield (N), CA3 subfield (O), and entire hippocampus (P). Compared to the GWI-VEH group, both nCUR groups displayed reduced percentages of IBA-1+ microglia expressing ED-1 (i.e., activated microglia) in the CA1 and CA3 subfields of the hippocampus and the entire hippocampus. Scale bar, A-L = 25 μm. **, p<0.01, and ***, p< 0.001; NS, not significant.

Moreover, we examined the effects of early nCUR therapy on microglial activation by quantifying the percentage of IBA-1+ microglia expressing ED-1 (CD68) in different subfields of the hippocampus. Examples of IBA-1+ microglia in the CA3 subfield of the hippocampus from different groups displaying morphology and ED-1 expression are illustrated in Fig. 4A-L. The morphology of microglia varied in different groups. Microglia in the naïve group displayed smaller soma and highly ramified processes (Fig. 4A), whereas the microglia in the GWI-VEH group exhibited hypertrophied soma and shorter processes with reduced ramifications (Fig. 4D). The morphology of microglia in GWI rats receiving nCUR, on the other hand, mostly resembled those in the naïve control group with highly ramified processes (Fig. 4G, J). Next, we compared percentages of activated microglia in different subfields of the hippocampus between the GWI-VEH group and GWI rats receiving 10 or 20 mg/Kg nCUR (Fig. 4M-P). The differences between groups were significant for the CA1 and CA3 subfields and the entire hippocampus (F=8.4-31.6, p<0.01-0.0001, Fig. 4N-P). Thus, nCUR treatment at both doses reduced reactive astrocytes and activated microglia, suggesting that the anti-inflammatory effects of nCUR involved modulation of astrocytes and microglia.

**Figure 5.**
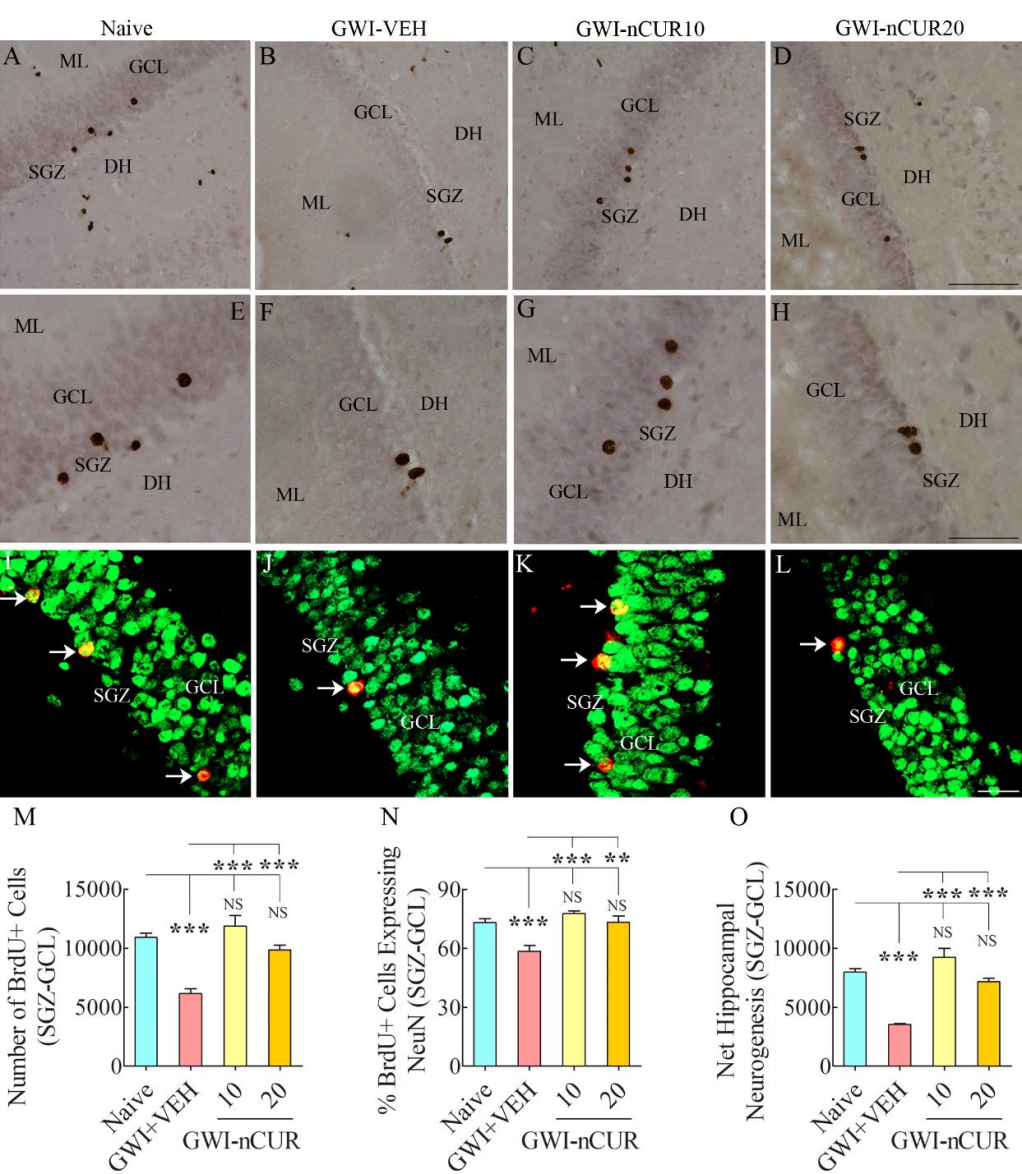
Five weeks of nCUR treatment, commencing 2 months after exposure to GWI-related chemicals and stress enhanced neurogenesis in the hippocampus. Figures in (A-H) show examples of 5’-bromodeoxyuridine-positive (BrdU+) cells in the SGZ-GCL of the hippocampus from naïve (A) and GWI rats receiving vehicle (GWI-VEH; B) or different doses of nCUR (GWI-nCUR10, C; GWI-nCUR20; D). (E, F, G, and H) are magnified views of regions from A, B, C, and D. Bar chart M compares the number of BrdU+ newly born cells in the SGZ-GCL of the hippocampus between different groups of rats. Note a significantly reduced number of BrdU+ cells in the GWI-VEH group compared to the naive control group. nCUR treatment at both doses enhanced the production of new cells (i.e., BrdU+ cells). Figures in I-L illustrate examples of BrdU+ cells expressing neuron-specific nuclear antigen (NeuN, arrows) from a naive control rat (I), a GWI rat receiving vehicle (J), a GWI rat receiving 10 mg/Kg nCUR (K), and a GWI rat receiving 20 mg/kg nCUR (L). The bar chart in N compares percentages of BrdU+ cells expressing NeuN, whereas the bar chart in (O) compares net hippocampal neurogenesis between different groups. Note significantly reduced percentage of BrdU+ cells expressing NeuN and net hippocampal neurogenesis in the GWI-VEH group compared to the naïve group. nCUR treatment at both doses normalized the neuronal differentiation of BrdU+ cells and net neurogenesis to naïve control levels. **, p < 0.01; ***, p<0.001; NS, not significant. DH, dentate hilus; GCL, granule cell layer; ML, molecular layer; SGZ, subgranular zone. Scale bar, A-D = 100 µm; E-H = 50 µm; I-L = 25 µm.

### Early nCUR treatment improved hippocampal neurogenesis in GWI rats:

We measured numbers of new cells and neurons born in the 5th week of nCUR/VEH treatment (equivalent to ~3 months after exposure to chemicals and stress) and survived for 3 weeks, using BrdU labeling. Examples of newly born cells expressing BrdU and newly born neurons expressing BrdU and NeuN in the SGZ-GCL of different groups are illustrated (Fig. 5A-L). Analyses revealed differences between groups when data such as the production of newly born cells, neuronal differentiation of newly born cells, and net neurogenesis were compared (F=10.8-30.5, p<0.001-0.0001, Fig. 5M-O). The animals in the GWI-VEH group demonstrated decreased newly born cell number, reduced neuronal differentiation of newly born cells, and diminished net neurogenesis compared to the naïve control group (p<0.001, Fig. 5M-O). Remarkably, nCUR treatment at both doses normalized all three measures of neurogenesis to naïve control levels (p>0.05). In comparison to the GWI-VEH group, the GWI-nCUR group displayed higher levels of newly born cells, neurons and neurogenesis (p<0.01-0.0001, Fig. 5M-O). Thus, early nCUR intervention for 5 weeks was sufficient for enhancing hippocampal neurogenesis in GWI rats.

**Figure 6.**
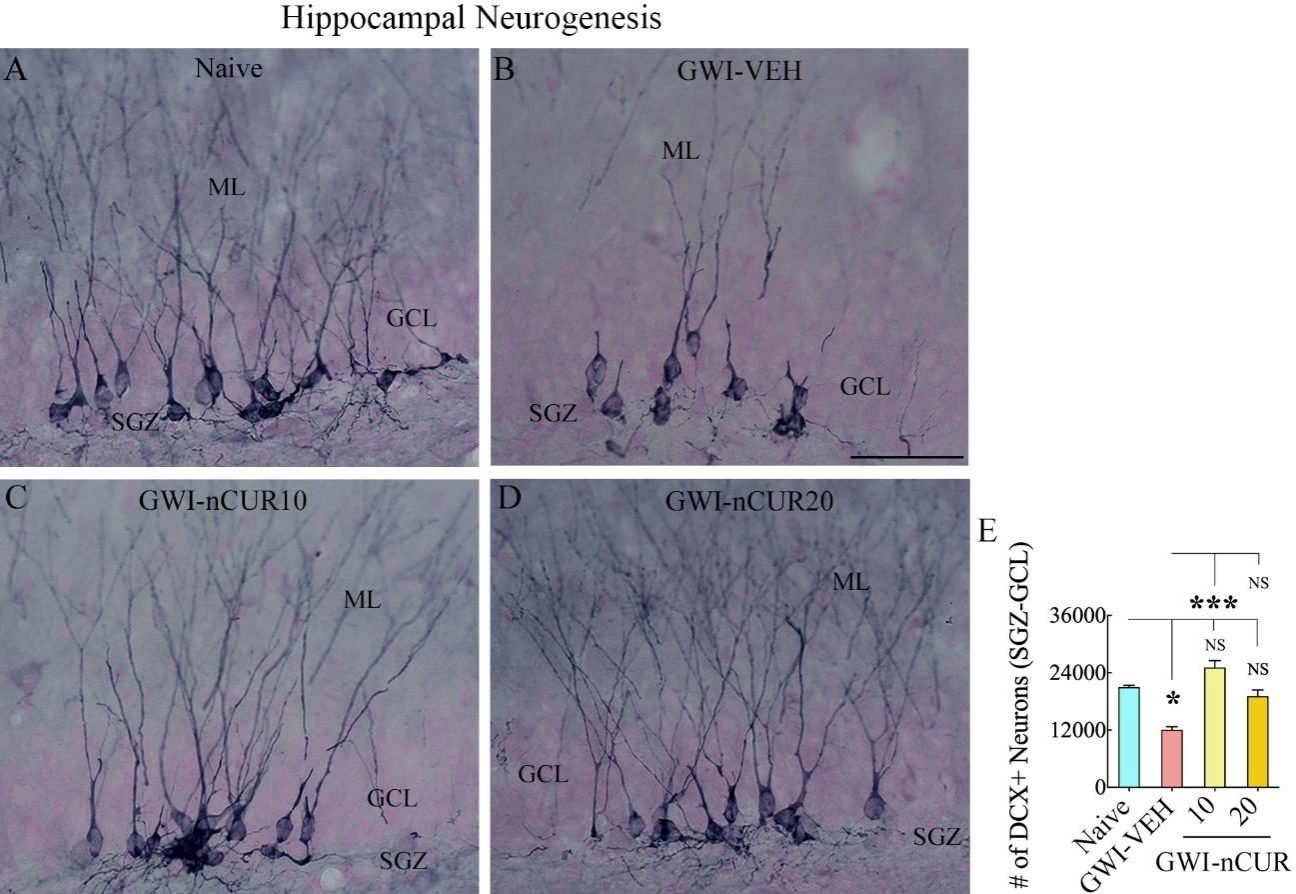
Eight weeks of nCUR treatment, commencing 2 months after exposure to GWI-related chemicals and stress, enhanced neurogenesis in the hippocampus. Figures A-D illustrate the distribution of doublecortin-positive (DCX+) newly born neurons from animals belonging to naïve (A), GWI-VEH (B), GWI-nCUR10 (C), and GWI-nCUR20 (D) groups. The bar chart E compares the number of DCX + neurons across different groups. Note that GWI rats receiving nCUR exhibited improved hippocampal neurogenesis. GCL, granule cell layer; ML, molecular layer; SGZ, subgranular zone. Scale bar, A-D = 50 μm. *, p<0.05, and ***, p< 0.001; NS, not significant.

We also measured DCX+ newly born neurons in the SGZ-GCL of the hippocampus, which provided information on hippocampal neurogenesis's status at the end of eight weeks of the vehicle or nCUR treatment. The morphology and distribution of DCX+ neurons in the SGZ-GCL of different groups are illustrated (Fig. 6A-D). Analyses revealed significant differences between groups (H=16.8, p<0.001, Fig. 6E). The animals in the GWI-VEH group showed a decreased number of DCX+ newly born neurons than the naïve group (p<0.05). Remarkably, nCUR at both doses normalized neurogenesis to naïve control levels (p>0.05). nCUR treatment at 10mg/kg dose considerably enhanced the number of DCX+ newly born neurons compared to the GWI-VEH group (p<0.001, Fig. 6E). Thus, early nCUR intervention for 8 weeks considerably enhanced hippocampal neurogenesis in GWI rats.

**Figure 7.**
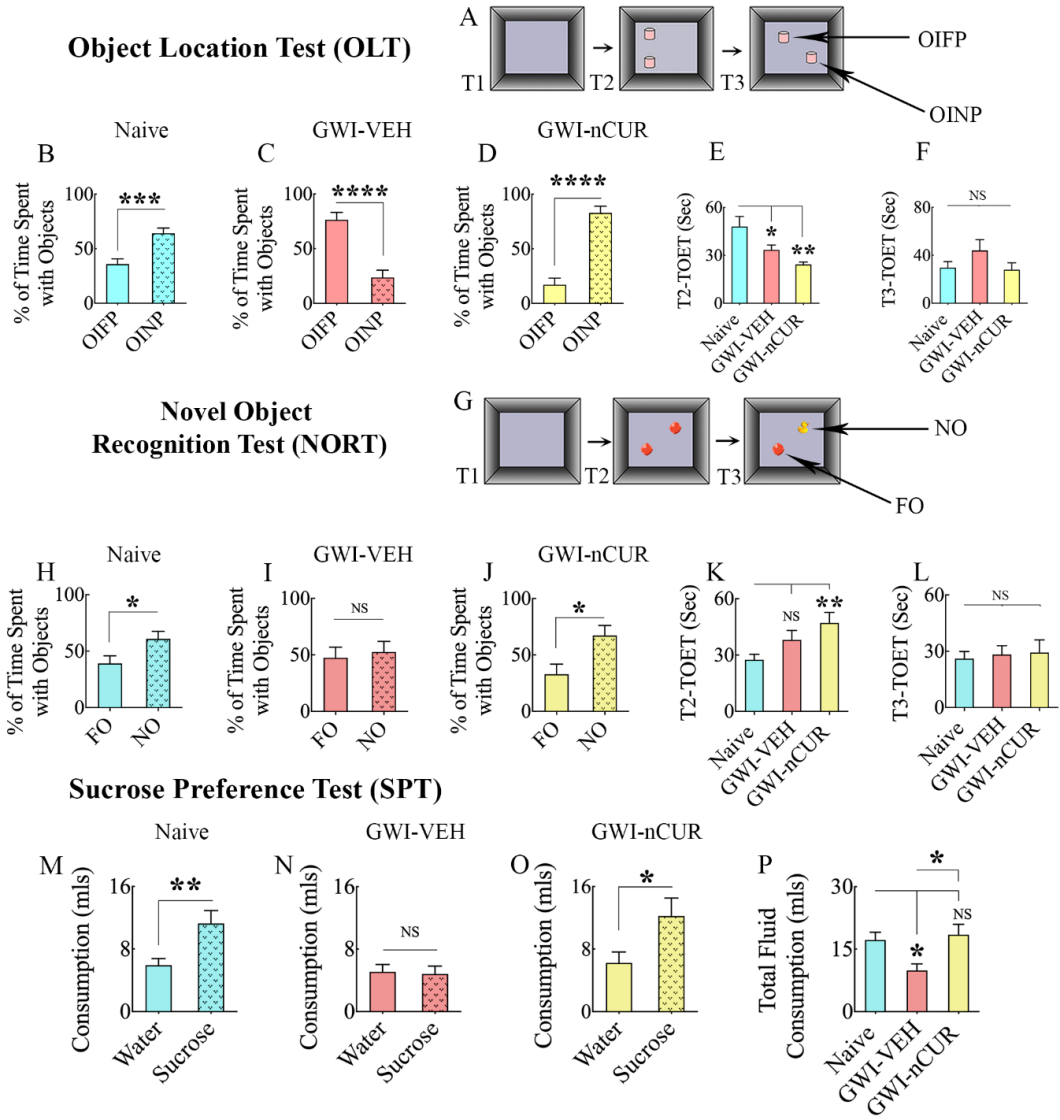
Twelve weeks of nCUR treatment in rats with chronic GWI improved cognitive and mood function. (A) shows the various phases involved in an object location test (OLT) whereas, the bar charts in B-D show the performance of animals belonging to naïve and GWI rats receiving vehicle (GWI-VEH) or nCUR (GWI-nCUR). The animals in the naïve group (B) preferred the object in a novel place (OINP) over the object in a familiar place (OIFP), implying their ability to recognize minor changes in the environment. Animals in the GWI-VEH group (C) were impaired, as they preferred the OIFP over the OINP, whereas animals in the nCUR group (D) behaved similarly to the naive control group, implying an improved cognitive function. The bar charts E-F compare the total object exploration times (TOETs) in trial-2 (T2) and T3. Cartoon (G) shows the various phases involved in a novel object recognition test (NORT), whereas the bar charts in H-J show the performance of animals belonging to different groups. The animals in the naïve group (H) preferred the novel object (NO) over the familiar object (FO), suggesting their proficiency for object recognition memory. Animals in the GWI-VEH group (I) were impaired, as they did not prefer the NO, whereas animals in the nCUR10 group (J) behaved akin to the naive control group, suggesting an improved recognition memory function. The bar charts K-L compare the TOETs in T2 and T3. The bar charts in the last row (M-P) show the results of a sucrose preference test. Animals in the naïve group (M) preferred sucrose-containing water over the standard water. Animals in the GWI-VEH group (N) exhibited anhedonia as they did not prefer the sucrose-containing water, whereas animals in the GWI-nCUR group preferred to drink the sucrose-containing water (O), implying no anhedonia. The bar chart in P compares the total fluid consumption across groups. *, p<0.05, **, p<0.01, ***, p<0.001, and ****, p<0.0001; NS, not significant.

**Figure 8.**
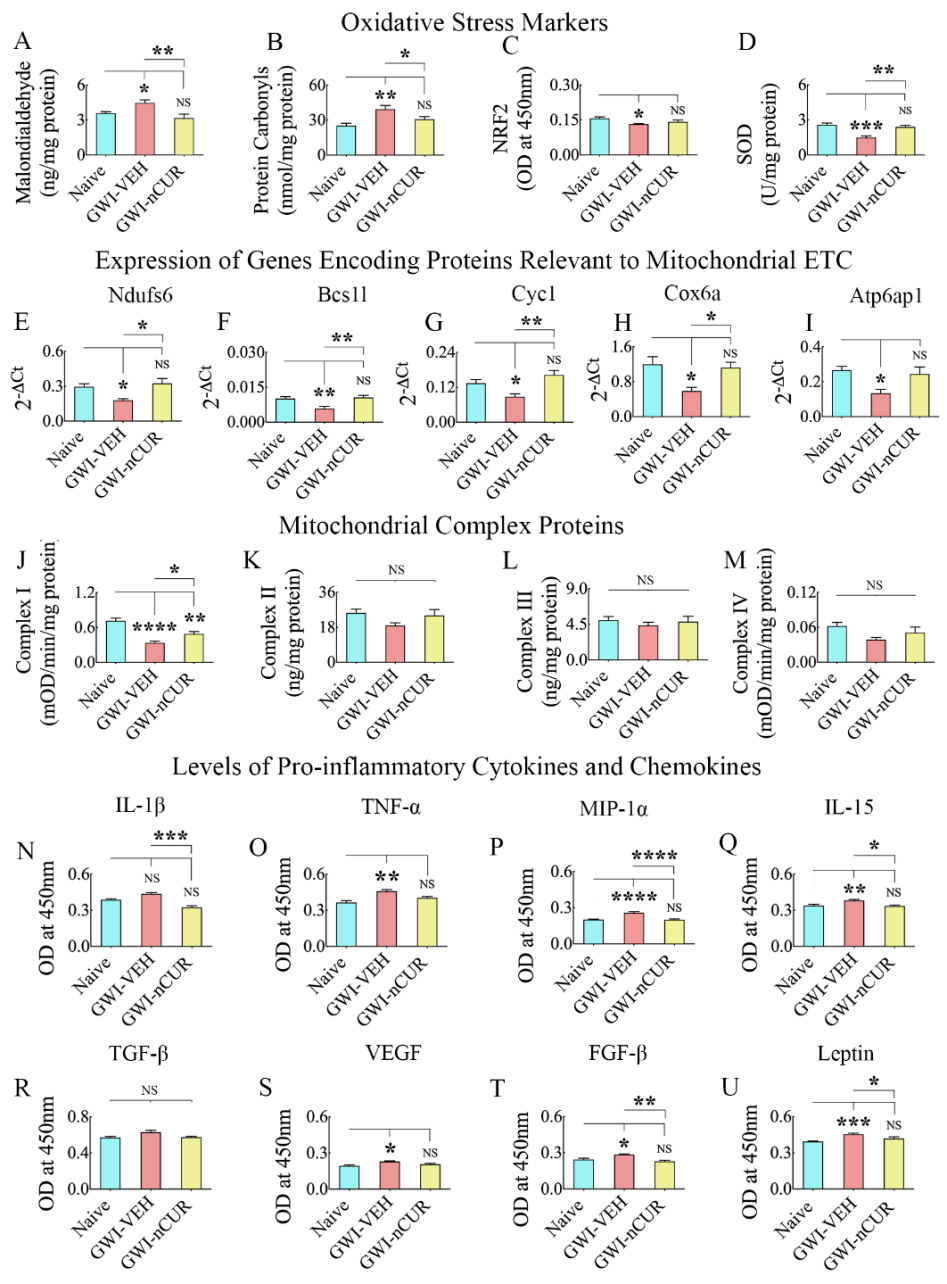
Twelve weeks of nCUR treatment in rats with chronic GWI reduced oxidative stress, improved mitochondrial function, and repressed proinflammatory cytokines and chemokines in the hippocampus. The bar charts in A-D compare the concentration of malondialdehyde (MDA; A), protein carbonyls (PCs; B), the nuclear factor erythroid 2-related factor 2 (NRF2; C), and superoxide dismutase (SOD; D) in the hippocampus between naïve, and GWI rats receiving vehicle (GWI-VEH) or nCUR (GWI-nCUR). Note that MDA and PCs were upregulated, and NRF2 and SOD were downregulated in the GWI-VEH group, compared to the naïve control group. In the GWI-nCUR, all four markers were normalized to naïve control levels. The bar charts E-I compare the expression of genes Ndufs6 (E), Bcs1l (F), Cyc1 (G), Cox6a (H), and Atp6ap1 (I) between different groups. These genes' expression was downregulated in the GWI-VEH group but normalized to naïve control levels in the GWI-nCUR group. The bar charts in J-M compare the concentration of mitochondrial complexes I-IV in the hippocampus between different groups. The bar charts in N-U compare the concentration of interleukin-1 beta (IL-1β), tumor necrosis factor-alpha (TNF-α), macrophage inflammatory protein 1 alpha (MIP-1α or CCL3), interleukin-15 (IL-15), transforming growth factor-beta (TGF-β), vascular endothelial growth factor (VEGF), fibroblast growth factor-beta (FGF-β) and Leptin in the hippocampus between different groups. All of these cytokines and chemokines except IL-1β and TGF-β were upregulated in the GWI-VEH group but significantly reduced in the GWI-nCUR group. *, p<0.05, **, p<0.01, ***, p<0.001, and ****, p<0.0001; NS, not significant.

### B. Effects of delayed nCUR intervention after exposure to GWI-related chemicals and stress

We next investigated the efficacy of 12 weeks of nCUR treatment (10 mg/Kg) in GWI animals, starting 8 months after the exposure to chemicals and stress. Since both doses (10 or 20 mg/Kg) of nCUR were equally efficacious for improving brain function in the early intervention study, we chose the lower dose for the delayed intervention study. Furthermore, a delayed nCUR intervention paradigm was chosen to simulate the treatment of chronic GWI in veterans. The 8-month time point after exposure to GWI-chemicals and stress in rats is equivalent to ~24 years of survival after exposure in humans [45].

### nCUR therapy improved cognitive, memory, and mood function in rats with chronic GWI

We first measured the competence for discerning minor changes in the environment using an OLT (Fig. 7A). The majority of animals (10-13/group from 12-16 tested/group) met the criteria employed for this task (i.e., exploration of objects ≥16 seconds in T2 and ≥8 seconds in T3). Proficiency for object location memory was intact in the age-matched naïve control animals (t=4.2, p<0.001, Fig. 7B) but was impaired in GWI animals receiving vehicle (t=5.6, p<0.0001, Fig. 7C). However, GWI animals receiving nCUR displayed no cognitive impairment (t=6.8, p<0.0001, Fig. 7D). Such changes were evident from animals in the naïve control and GWI-nCUR groups preferring the OINP over the OIFP (Fig. 7B, D], and animals in the GWI-VEH group displaying a higher propensity to explore the OIFP (Fig. 7C). The TOETs in T2 differed between groups (F=6.9, p<0.01) but not in T3 (H=2.1, p>0.05, Fig. 7E-F). However, the average object exploration time in each group was >25 seconds in both T2 and T3. Next, we determined the recognition memory function using a NORT (Fig. 7G), which uncovered the proficiency for discriminating the NO from the FO. The majority of animals (10-11/group from 13-16 tested/group) met the criteria employed for this task (i.e., exploration of objects ≥16 seconds in T2 and ≥8 seconds in T3). Adeptness for recognition memory was intact in the naïve control group (t=2.3, p<0.05, Fig. 7H), but impaired in the GWI-VEH group (t=0.4, p>0.05, Fig. 7I). However, animals in the GWI-nCUR presented no recognition memory impairment (t=2.7, p<0.05, Fig. 7J). Such alterations were evident from animals in the naïve control and GWI-nCUR groups preferring the NO over the FO (Fig. 7H, J), and animals in the GWI-VEH group showing no preference for either NO or FO (Fig. 7I). The TOETs in T2 differed between groups (H=9.4, p<0.01) but not in T3 (H=0.05, p>0.05, Fig. 7K-L). However, the average object exploration time in each group was >26 seconds in both T2 and T3.

Furthermore, investigation of mood function using an SPT (n=11-15/group) demonstrated no anhedonia in the naïve control group, but animals in the GWI-VEH group displayed significant anhedonia (Fig. 7M-N). Remarkably, anhedonia was reversed in GWI animals receiving nCUR (Fig. 7O). Lack of anhedonia in naïve and GWI-nCUR groups was evident from their preference to drink higher amounts of sucrose-containing water than regular water (Naïve, Mann-Whitney U=34.5, GWI-nCUR, t=2.2, p<0.05-0.01, Fig. 7M, O) whereas the presence of anhedonia in the GWI-VEH group was apparent from the lack of propensity to drink sweet water over regular water (t=0.2, p>0.05, Fig. 7N). The overall fluid consumption varied significantly between groups (F=4.6, p<0.05, Fig. 7P). Compared to the naïve group, the consumption was less in the GWI-VEH group (p<0.05) but comparable in the GWI-nCUR group. Thus, 10 mg/Kg dose of nCUR treatment for 12 weeks was efficacious for improving cognitive, recognition memory, and mood function in GWI rats, even when the treatment commenced 8 months after exposure to GWI related chemicals and stress.

### nCUR therapy eased OS, and hypoactive mitochondria in rats with chronic GWI

We measured OS markers (MDA and PCs, Nrf2, and SOD), the expression of genes encoding proteins relevant to the mitochondrial electron transport chain (ETC), and mitochondrial complex proteins (complex I, II, III, and IV) to determine the efficacy of nCUR treatment in reducing OS in chronic GWI. Evaluation showed differences between groups for MDA, PCs, Nrf2, and SOD (F/H=6.4-13.2, p<0.05-0.001, Fig. 8A-D). The concentration of MDA and PCs were higher in the GWI-VEH group, in comparison to the naïve control group (p<0.05-0.01), but were normalized to control levels in the GWI-nCUR group (p>0.05 versus the naïve control group, and p<0.05-0.01 versus the GWI-VEH group, Fig. 8A-B). The concentration of the master regulator of OS, Nrf2 and the antioxidant SOD, was reduced in the GWI-VEH group (p<0.05-0.001) but normalized to naïve control levels in the GWI-nCUR group (p>0.05, Fig. 8C-D). Significant differences between groups were also seen for many genes encoding proteins relevant to the mitochondrial ETC, which include *Ndufs6, Bcs1L, Cyc1, Cox6a, Atp6ap1* (F/H=4.8-8.5, p<0.05-0.01, Fig. 8E-I). The expression of these genes was reduced in the GWI-VEH group (p<0.05-0.01) but brought to naïve control levels in the GWI-nCUR groups (p>0.05, Fig. 8E-I). The expressions of other genes measured (*Cox4i2, Ndufs7, SdhB, Slc25a10, SdhA*) also showed a similar trend but were not significant statistically (data not illustrated). The activity of mitochondrial complex I showed differences between groups (F=19.3, p<0.0001, Fig. 8J). The GWI-VEH group displayed considerably reduced complex I activity compared to the naïve control group (p<0.0001, Fig. 8J). The activities of complex II showed a similar trend but analysis did not show differences between groups (F=2, p>0.05, Fig. 8K. Thus, elevated levels of OS markers, decreased Nrf2 and SOD, diminished expression of many genes encoding proteins relevant mitochondrial ETC, and reduced complex I and IV activity in the GWI-VEH group suggest hypoactive mitochondria at ~11 months after exposure to GWI-related chemicals and stress. Remarkably, nCUR therapy normalized all alterations linked to OS and mitochondrial function.

**Figure 9.**
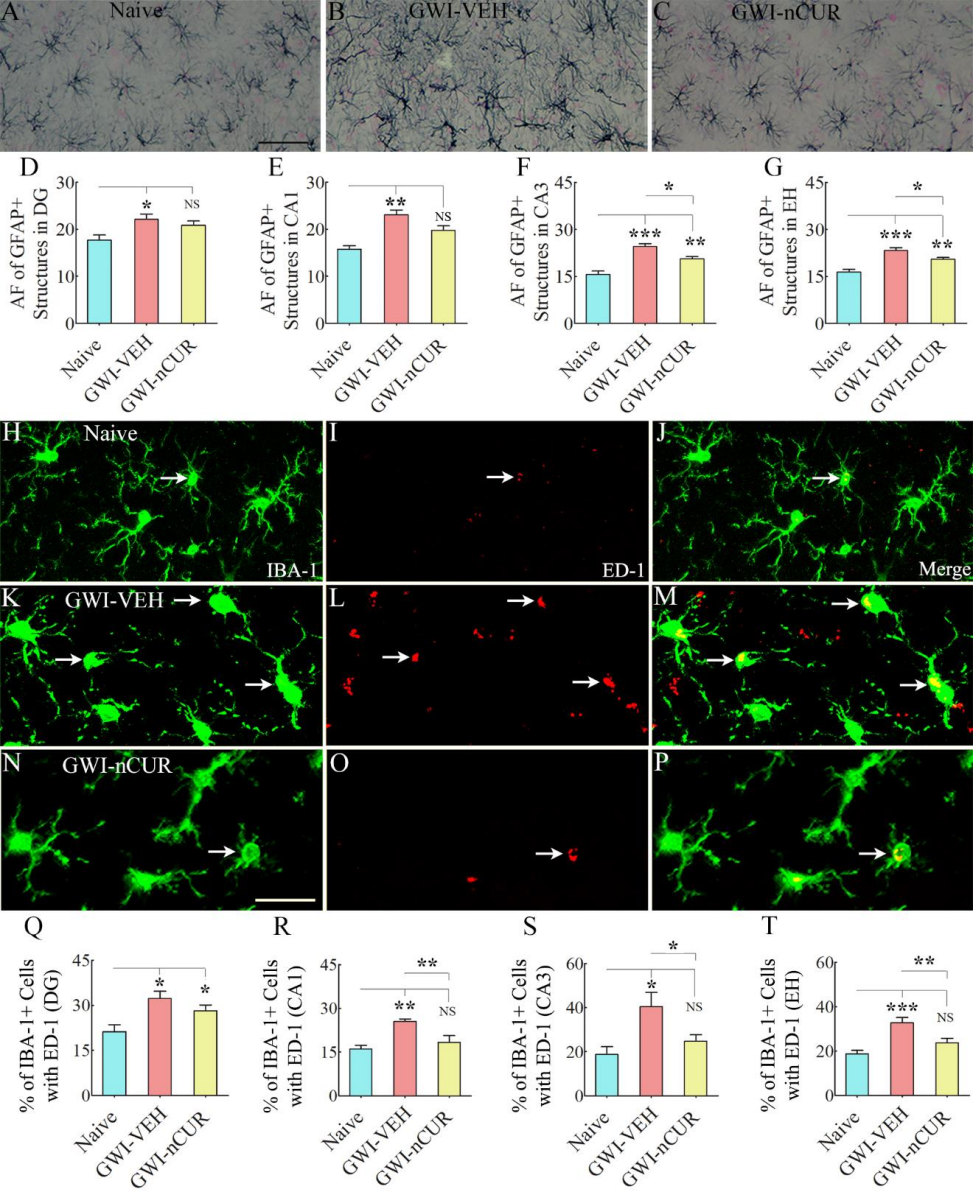
Twelve weeks of nCUR treatment in rats with chronic GWI reduced astrocyte hypertrophy and activated microglia in the hippocampus. Figures A-C show examples of GFAP+ astrocytes from the CA3 subfield of naïve control (A), GWI-VEH (B), GWI-nCUR (C) groups. The bar charts D-G compare the area fraction (AF) of GFAP+ structures in the DG (D), the CA1 subfield (E), the CA3 subfield (F), and the entire hippocampus (G) between different groups. AF’s of GFAP+ structures were higher in the GWI-VEH group in all hippocampal regions but significantly reduced in the CA1 and CA3 subfields and the entire hippocampus in the GWI-nCUR group. Figures H-P illustrate examples of IBA-1+ microglia displaying ED-1+ structures (activated microglia) from the CA3 subfield of naïve control (H-J), GWI-VEH (K-M), GWI-nCUR (N-P) groups. The bar charts Q-T compare percentages of IBA-1+ microglia with ED-1 in the DG (Q), the CA1 subfield (R), the CA3 subfield (S), and the entire hippocampus (T) between different groups. The percentages of activated microglia were higher in the GWI-VEH group in all hippocampal regions but significantly reduced in the CA1 and CA3 subfields and the entire hippocampus in the GWI-nCUR group. Scale bar, A-C = 50 μm; H-P = 25 μm; *, p< 0.05, **, p<0.01, and ***, p<0.001; NS, not significant.

### nCUR therapy reduced proinflammatory cytokines and chemokine levels, reactive astrocytes and activated microglia in the hippocampus of rats with chronic GWI

To discern the effects of delayed nCUR intervention on neuroinflammation in the hippocampus of rats with chronic GWI, we measured multiple proinflammatory cytokines and chemokines, astrocyte hypertrophy, and activated microglia. The cytokine array showed differences between groups for IL-1β, TNF-α, Mip-1α, IL-15, TGF-β, VEGF, FGF-β, and leptin (F/H=4.5-23.8). The differences were significant for TNF-α, Mip-1α, IL-15, VEGF, FGF-β, and leptin (p<0.05-0.0001, Fig. 8O-Q, S-U). The levels of these proinflammatory mediators were elevated in the GWI-VEH group (p<0.05-0.0001) but were normalized to naïve control levels in the GWI-nCUR group (p>0.05, Fig. 8O-Q, S-U).

Analysis of GFAP+ astrocytes revealed astrocyte hypertrophy in all hippocampal subfields compared to the naïve control group, but animals that received nCUR displayed reduced astrocyte hypertrophy. Examples of GFAP+ astrocytes in the CA3 subfield of the hippocampus from different groups are presented (Fig. 9A-C). Comparison of area fraction of GFAP+ astrocytic elements showed significant differences between groups for individual subfields of the hippocampus and when the hippocampus was taken in its entirety (F/H=4.6-21.4, p<0.05-0.0001, Fig. 9D-G). Increased density of hypertrophied astrocytes was observed in animals belonging to the GWI-VEH group compared to the naïve control group (p<0.05-0.001, Fig. 9D-G). In DG and CA1 subfields, astrocyte hypertrophy was normalized to naïve control levels (p>0.05, Fig. 9D-E). In CA3 subfield and when the hippocampus was taken in entirety, the area fraction of astrocytes in the GWI-nCUR group was reduced compared to the GWI-VEH group (p<0.05, Fig. 9F-G). Furthermore, analyses of the percentage of IBA-1+ microglia expressing ED-1 (Fig. 9H-P) showed differences between groups in all subfields of the hippocampus and when the entire hippocampus was taken in entirety (F=6.0-12.3, p<0.05-0.01, Fig. 9Q-T). Higher percentages of activated microglia were observed in animals belonging to the GWI-VEH group compared to the naïve control group (p<0.05-0.001, Fig. 9Q-T). In CA1 and CA3 subfields and the entire hippocampus, the percentages in the GWI-nCUR group was reduced compared to the GWI-VEH group (p<0.05-0.01) and normalized to naïve control levels (p>0.05, Fig. 9R-T). Thus, nCUR treatment reduced reactive astrocytes and activated microglia, suggesting that the antiinflammatory effects of nCUR involved modulation of astrocytes and microglia. Thus, nCUR treatment was efficacious for considerably alleviating neuroinflammation in the hippocampus of rats with chronic GWI.

### nCUR therapy reduced NLRP3 inflammasomes in microglia of rats with chronic GWI

**Figure 10.**
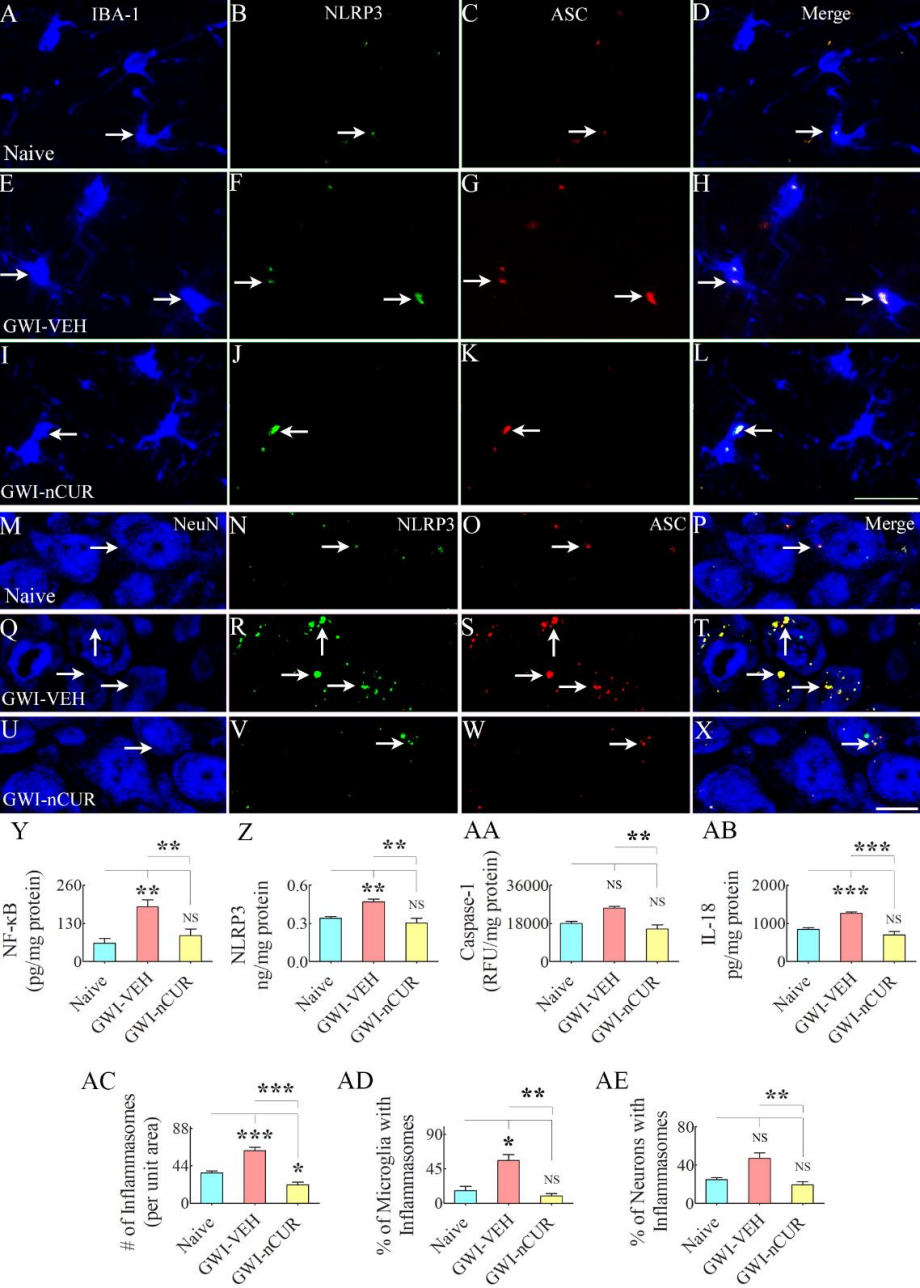
Twelve weeks of nCUR treatment in rats with chronic GWI reduced NLRP3 inflammasome formation in the hippocampus. Figures A-X illustrate examples of NLRP3 inflammasomes in IBA-1+ microglia (A-L) and NeuN+ neurons (M-X) from the CA3 subfield of naïve control (A-D, M-P), GWI-VEH (E-H, Q-T), GWI-nCUR (I-L, U-X) groups. The bar charts in Y-AE compare concentrations of NF-kB (Y), NLRP3 (Z), caspase-1 (AA), and IL-18 (AB), the number of inflammasomes/unit area (AC), the percentage of microglia with inflammasomes (AD), and the percentage of neurons with inflammasomes (AE). All of these measures except caspase 1 and the percentage of inflammasomes in neurons were upregulated in the GWI-VEH group but significantly reduced in the GWI-nCUR group. Scale bar, A-L = 25 μm; M-X = 12.5 μm; *, p<0.05, **, p<0.01, ***, p<0.001; NS, not significant.

**Figure 11.**
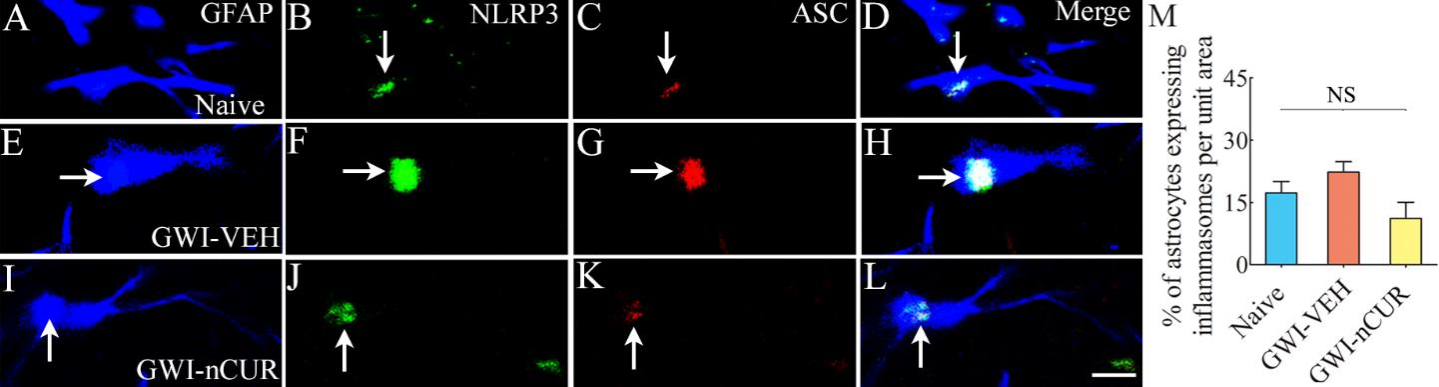
NLRP3 inflammasomes in astrocytes belonging to different groups. Figures A-L illustrate examples of NLRP3 inflammasomes in GFAP+ astrocytes from the CA3 subfield of naïve control (A-D), GWI-VEH (E-H), GWI-nCUR (I-L) groups. Bar chart M compares the percentage of astrocytes expressing NLRP3 inflammasomes between different groups. Note that no significant difference was observed in the percentage of astrocytes expressing inflammasomes across the three groups. Scale bar, A-L = 6.25 μm. NS, not significant.

We quantified the extent of NLRP3 inflammasomes in the hippocampus to understand the potential mechanisms by which nCUR alleviated neuroinflammation. Examples of microglia and neurons displaying NLRP3 inflammasomes in different groups are illustrated (Fig. 10A-X). We first quantified the concentrations of NF-kB, NLRP3, caspase-1 and IL-18, all of which showed differences between groups (F/H=9-18.5, p<0.01-0.0001, Fig. 10Y-AB). NF-kB, NLRP3, and IL-18 were elevated in the GWI-VEH group compared to the naïve control group (p<0.01-0.001). In the GWI-nCUR group, NF-kB, NLRP3, and IL-18 concentrations were normalized to naïve control levels (p>0.05, Fig. 10Y-Z, AB). Also, the GWI-nCUR group displayed less caspase 1 than the GWI-VEH group (p<0.01, Fig. 10AA). We next measured the number of inflammasomes (i.e., particles expressing NLRP3 and ASC per unit area of the CA3 subfield, which also showed differences between groups (F=36.1, p<0.0001, Fig. 10AC. The particles co-expressing NLRP3 and ASC were increased in the GWI-VEH group (p<0.001) but reduced to below naïve control levels in the GWI-nCUR group (p<0.05, Fig. 10AC). Furthermore, we quantified percentages of microglia, neurons and astrocytes displaying inflammasomes via triple immunofluorescence for NLRP3 and ASC with markers of microglia (IBA-1), neurons (NeuN) or astrocytes (GFAP), which showed differences between groups for microglia (H=11.7, p<0.001, Fig. 10AD). Significantly higher percentages of microglia displayed inflammasomes in the GWI-VEH group than the naïve control group (p<0.05, Fig. 10A-L, AD). Remarkably, inflammasomes in microglia were back to naïve control levels in GWI-nCUR group (p>0.05, Fig. 10AD). Neurons and astrocytes showed a similar trend, but the differences were not significant statistically (Fig. 10M-X, AE and Fig. 11). Thus, nCUR treatment was efficacious for restraining the inflammasome formation in the hippocampus of rats with chronic GWI.

### Rats with chronic GWI displayed enhanced hippocampal neurogenesis with 12 weeks of nCUR treatment

**Figure 12.**
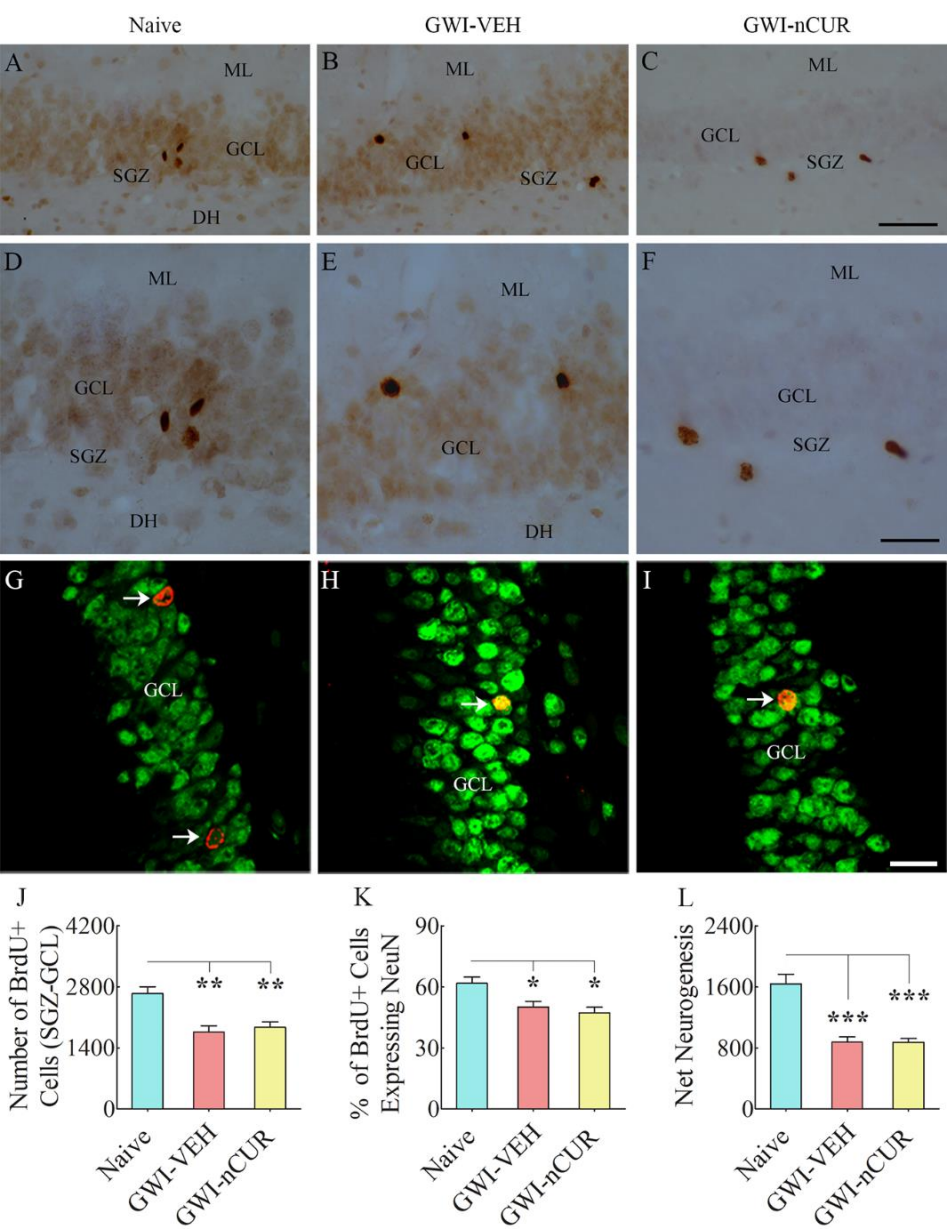
Seven weeks of nCUR treatment in rats with chronic GWI did not improve neurogenesis in the hippocampus. Figures in A-F illustrate examples of 5’-bromodeoxyuridine-positive (BrdU+) cells in the SGZ-GCL of the hippocampus from naïve (A) and GWI rats receiving vehicle (GWI-VEH; B) or nCUR (GWI-nCUR, C). D, E, and F are the magnified views of regions from A, B, and C. The bar chart J compares the number of BrdU+ newly born cells in the SGZ-GCL of the hippocampus between different groups of rats. Note a significantly reduced number of BrdU+ cells in the GWI-VEH group compared to the naive control group. nCUR treatment did not improve the number of BrdU+ newly born cells. Figures in G-I illustrate examples of BrdU+ cells expressing neuron-specific nuclear antigen (NeuN, arrows) from a naive control rat (G), a GWI rat receiving vehicle (H), and a GWI rat receiving nCUR (I). The bar chart K compares percentages of BrdU+ cells expressing NeuN, whereas the bar chart L compares net hippocampal neurogenesis between different groups. Note significantly reduced percentage of BrdU+ cells expressing NeuN and net hippocampal neurogenesis in the GWI-VEH group compared to the naïve group. nCUR treatment did not improve either the percentage of BrdU+ cells expressing NeuN or net neurogenesis. *, p < 0.05; **, p < 0.01; ***, p<0.001. ML, molecular level; GCL, granule cell layer; SGZ, subgranular zone. Scale bar, A-C = 100 µm; D-F = 50 µm; G-I = 25 µm.

**Figure 13.**
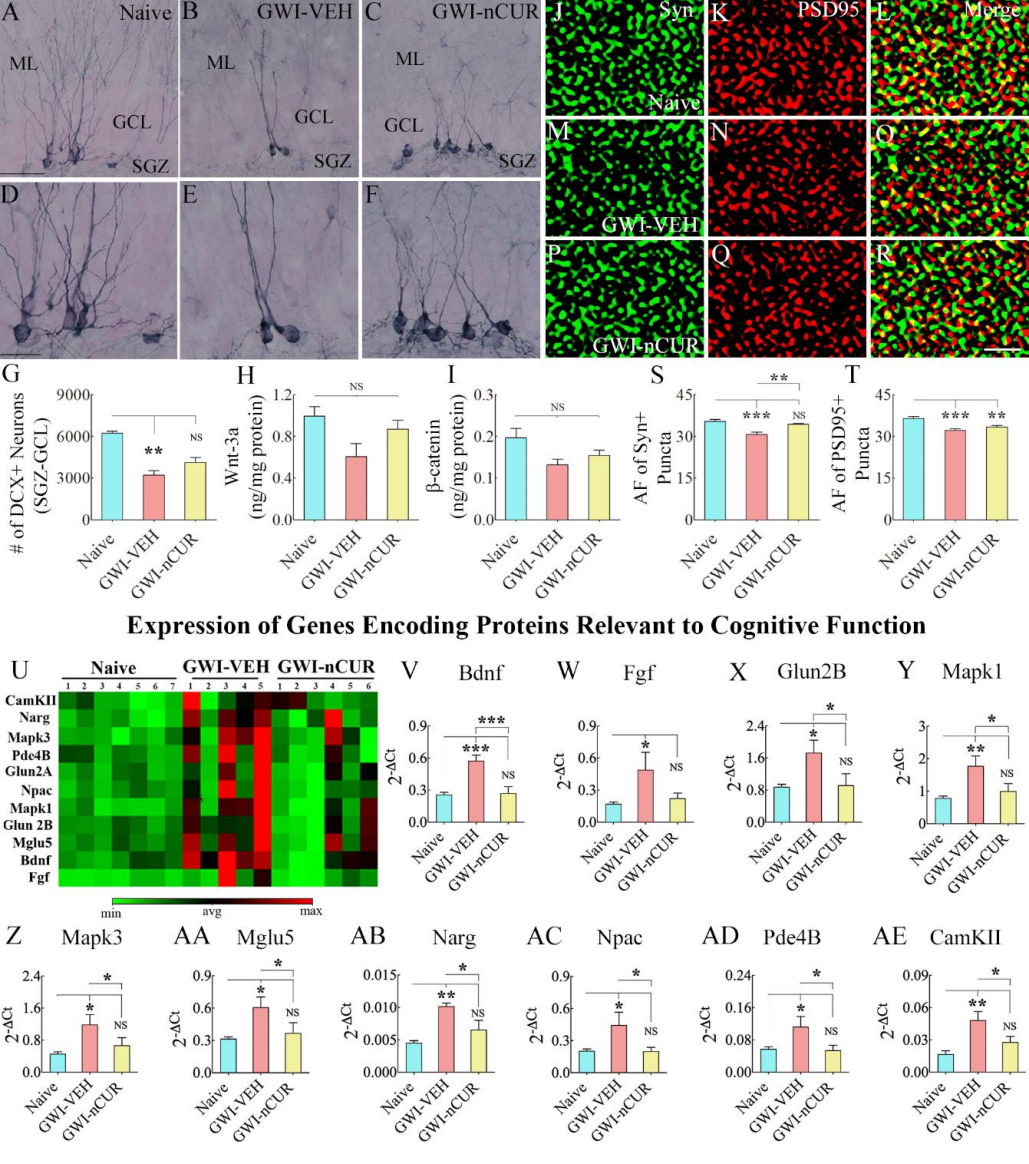
Twelve weeks of nCUR treatment in rats with chronic GWI enhanced neurogenesis, reduced synapse loss, and stabilized expression of genes involved in altered cognitive function in the hippocampus. Figures A-F illustrate examples of doublecortin-positive (DCX+) newly born neurons from naïve control (A), GWI-VEH (B), GWI-nCUR (C) groups. D, E, and F are magnified views of regions from A, B, and C. The bar chart G compares the number of DCX+ neurons between different groups. Neurogenesis was decreased in the GWI-VEH group but significantly increased in the GWI-nCUR group. GCL, granule cell layer; ML, molecular layer; SGZ, subgranular zone. Scale bar, A-C = 50 μm; D-F = 25 μm. The bar charts H and I compare Wnt-3a (H) and beta-catenin (I) concentration between different groups. Figures J-R shows examples of synaptophysin+ (Syn+) and postsynaptic density 95+ (PSD95+) puncta in the dentate molecular layer from naïve control (J-L), GWI-VEH (M-O), GWI-nCUR (P-R) groups. The bar charts S-T compare area fraction (AF) of Syn+ and PSD95+ puncta in the dentate molecular layer between different groups. Both Syn+ and PSD95+ puncta were reduced in the GWI-VEH group, and Syn+ puncta were normalized to the naïve control level in the GWI-nCUR group. Scale bar, J-R = 1 μm. Figure U compares the relative expression of various genes involved in altered cognitive function between different groups. The bar charts in V-AE compare the expression of Bdnf (V), Fgf (W), Glun2B (X), MapK1 (Y), MapK3 (Z), Mglu5 (AA), Narg (AB), Npac (AC), Pde4B (AD), and CamKII (AE) between different groups. All genes exhibited increased expression in the GWI-VEH group but were normalized to the naïve control level in the GWI-nCUR group. *, p<0.05, **, p<0.01, and ***, p<0.001; NS, not significant. DH, dentate hilus; GCL, granule cell layer; ML, molecular layer; SGZ, subgranular zone.

We first measured numbers of new cells and neurons born in the 7^th^ week of nCUR/VEH treatment (equivalent to ~9.5 months after exposure to chemicals and stress) and survived for 6 weeks. The number of newly born cells, neuronal differentiation of newly born cells, and net neurogenesis were decreased in the GWI-VEH group compared to the naïve control group (F=6.8-24.8, p<0.05-0.0001, Fig. 12J-L). nCUR therapy did not enhance any of the neurogenesis measures (Fig. 12A-L). We next quantified DCX+ newly born neurons (Fig. 13A-F), which provided an index of the status of hippocampal neurogenesis at the end of 12 weeks of the vehicle or nCUR treatment. Significant differences were seen between groups (H=12.8, p<0.0001, Fig. 13G). The GWI-VEH group showed a decreased number of DCX+ newly born neurons than the naïve group (p<0.01). nCUR treatment normalized the number of DCX+ newly born neurons to the naive group level (p>0.05, Fig. 13G). Thus, nCUR therapy for 6 weeks was insufficient for enhancing neurogenesis in rats with chronic GWI. However, 12 weeks of nCUR treatment was efficacious for increasing neurogenesis in the chronic phase of GWI. We also measured Wnt-3a and beta-catenin levels in the hippocampus to understand the potential mechanisms by which nCUR enhanced neurogenesis. No significant differences were seen for Wnt-3a and beta-catenin (H=4.8-5.1, p>0.05, Fig. 13H-I).

### nCUR therapy to rats with chronic GWI reduced synapse loss and normalized the expression of genes encoding proteins important for pro-cognitive activity

Analyses of Syn+ and PSD95+ puncta in the outer molecular layer of the DG (Fig. 13J-R) revealed group differences (F=12.3-13.5, p<0.001, Fig. 13 [S-T]). The GWI-VEH group showed reduced area fractions of Syn+ and PSD95+ puncta compared to the naïve control group (p<0.001, Fig. 13S-T). In the GWI-nCUR group, the area fraction of Syn+ puncta was increased to naïve control levels with no changes in the area fraction of PSD95+ puncta. The results implied that nCUR therapy reduced synapse loss in the chronic phase of GWI. We next measured the expression of genes encoding proteins relevant to maintaining pro-cognitive effects (Fig. 13U). Group differences were seen for the expression of *Bdnf, Fgf, Glun2B, Mapk1, Mapk3, mglu5, Narg, Npac, Pde4B, CamKII genes* (F/H= 4.1-13.7, p<0.05-0.001, Fig. 13V-AE). The GWI-VEH group showed enhanced expression of all of these genes compared to the naïve group (p<0.05-0.001). nCUR treatment normalized the expression of these genes to naïve control levels (p>0.05, Fig. 13V-AE). Thus, nCUR therapy normalized the expression of genes encoding proteins relevant to sustaining pro-cognitive effects.

## DISCUSSION

This study provides new evidence that low-dose, intermittent, nCUR therapy is efficacious for alleviating cognitive and mood dysfunction in a rat model of GWI with treatment commencing at either early or a protracted timepoint after the exposure to GWI-related chemicals and stress. Better cognitive ability with nCUR therapy in GWI rats was apparent from their proficiency for recognizing minor changes in the immediate environment and novel object recognition memory, whereas improved mood function could be inferred from the reversal of anhedonia. Notably, enhanced brain function following nCUR therapy was concomitant with multiple favorable cellular and molecular alterations in the hippocampus of GWI rats, which comprised reduced OS, improved mitochondrial function, diminished neuroinflammation, enhanced neurogenesis, reduced synapse loss, and normalized expression of multiple genes encoding proteins germane to conserving normal cognitive and mood function

Unrelenting cognitive and mood impairments are among the most conspicuous symptoms in veterans afflicted with GWI [1, 57-59], which is associated with worsened functional connectivity between diverse networks in the sensorimotor domain [60]. Incessant cognitive and mood impairments are also the salient features of animal models of GWI [1, 14-16, 18, 22-25]. Brain tissue analyses at various time points after exposure to GWI-related chemicals and stress in the animal model employed in this study have identified many progressive neuropathological changes that likely contribute to cognitive and mood dysfunction in GWI. These include chronically elevated ROS levels, mitochondrial dysfunction, increased levels of multiple proinflammatory mediators, reactive astrocytes and activated microglia, and waned neurogenesis in the brain [15, 17, 23-25]. The other prototypes of GWI have also displayed comparable adverse neuropathological alterations [19, 21, 61-64]. Furthermore, many cellular and molecular changes observed in animal models of GWI could be corroborated with findings in veterans with GWI, which include increased OS [65-66] and chronic neuroinflammation [67-69]. We chose to test the efficacy of nCUR therapy in this study because of its ability to regulate ROS levels, improve mitochondrial function, repress the neuroinflammatory cascade, and improve neurogenesis through several mechanisms. Indeed, the low-dose nCUR therapy in this study improved cognitive and mood function in GWI rats with both early and delayed treatment paradigms. Conspicuously, better brain function at both timepoints was associated with reduced OS, improved mitochondrial function, suppressed neuroinflammation, and enhanced neurogenesis. The delayed intervention study results are particularly relevant to the potential nCUR treatment in veterans afflicted with GWI. The efficacy of nCUR therapy ascertained at 8 months after exposure to GWI-related chemicals and stress in this study is equivalent to testing at ~24 years after exposure to chemicals and stress in humans [45].

The proficiency of nCUR to restrain ROS levels in rats with GWI was evident from reduced concentrations of the lipid peroxidation marker MDA [70], protein oxidation marker PCs [71], and normalized expression of OS master regulator NRF2 [72] and the antioxidant SOD. The competence of nCUR to improve mitochondrial function in GWI rats could be gleaned from several observations. At 4 months after the exposure to GWI-related chemicals and stress, the animals displayed hyperactive mitochondria, which was evident from the enhanced activity of mitochondrial complexes I, II, and IV, consistent with the increased expression of multiple genes encoding proteins relevant to OS and mitochondrial ETC observed in our previous studies [15, 17, 24]. Eight-weeks of nCUR therapy commencing two months post-exposure normalized or significantly reduced the activity of mitochondrial complexes II, and IV. Conversely, the GWI animals exhibited mitochondrial hypofunction at 11 months post-exposure, which was typified by reduced expression of several mitochondria ETC-specific genes (*Ndufs6, Bcs1l, Cyc1, Cox6a*) and substantially reduced activity of complex I. These genes encode proteins contributing to the peripheral arm of complex I (*Ndufs6*) [73], the assembly of mitochondrial complex III (*Bcs1l*) [74], the heme-containing subunit of mitochondria complex III, and cellular energy production (*Cyc1*) [75], and complex IV assembly (*Cox6a*) [76]. Twelve-weeks of nCUR therapy commencing eight months post-exposure normalized the expression of all four genes. Notably, the waned *Ndufs6* expression was associated with complex I deficiency in GWI rats, but nCUR therapy normalized both gene expression and complex I activity. nCUR treatment also normalized a gene involved in the acidification of intracellular organelles (Atp6ap1) [77]. The transformation of hyperactive mitochondria at an earlier timepoint into mitochondrial hypofunction at an extended timepoint in GWI rats reflects the inability of mitochondria to sustain hyperactivity for extended periods, likely due to high ETC protein demands and the continued adverse effect of high ROS levels on mitochondrial function. Indeed, studies have shown that incessantly elevated ROS levels could cause mitochondrial DNA mutations, damage the mitochondrial ETC, alter membrane permeability, and influence Ca^2+^ homeostasis and mitochondrial defense systems [78]. Therefore, improved mitochondrial function in GWI rats receiving nCUR is likely mediated through antioxidant effects, apparent from reduced MDA and PCs and normalized NRF2 and SOD expression. Such a supposition is also harmonious with the previous studies demonstrating the ability of CUR to reduce OS through direct ROS scavenging [79] and by increasing antioxidant enzymes [80] and NRF2 [81]. Besides, CUR has mitochondria-protecting properties [82].

The capability of nCUR to restrain neuroinflammation in GWI rats was apparent from the normalized levels of proinflammatory markers, reduced astrocyte hypertrophy, and activated microglia. At 4 months after the exposure to GWI-related chemicals and stress, the animals displayed increased concentrations of TNF-α, astrocyte hypertrophy, and a higher density of activated microglia. Eight-weeks of nCUR therapy commencing two months post-exposure significantly reduced the TNF-α concentration, astrocyte hypertrophy, and activated microglia. At 11 months post-exposure, the animals displayed increased levels of TNF-α, Mip-1α, IL-15, VEGF, FGF-β, and leptin, astrocyte hypertrophy, and activated microglia. Twelve-weeks of nCUR therapy commencing eight months post-exposure normalized or significantly reduced the levels of all markers of neuroinflammation measured in this study. We also investigated the potential role of NLRP3 inflammasomes in maintaining chronic neuroinflammation in rats with chronic GWI. NLRP3 inflammasomes are multiprotein complexes in the cytoplasm that activate the proinflammatory caspase-1, eventually leading to proinflammatory maturation and secretion of IL-1β and IL-18 [83-84]. The formation of NLRP3 inflammasomes is triggered by danger-associated molecular patterns (DAMPs) in the CNS, and in increased OS conditions such as in chronic GWI, heme and metabolites could serve as DAMPs [85]. The NLRP3 inflammasome comprises NLR, ASC, and procaspase-1, with ASC functioning as an adaptor that links NLR with the procaspase-1 [84, 86]. The NLRP3 inflammasome formation sequentially involves NF-kB activation via DAMPs, the NF-kB-dependent transcriptional upregulation of NLRP3 and pro-IL-1β [87], and oligomerization and activation of the NLRP3 inflammasome [84]. In this study, active inflammasome formation in rats with chronic GWI could be confirmed from elevated NF-kB, NLRP3, and IL-18 levels. Furthermore, increased occurrence of inflammasomes could be visualized from NLRP3-ASC complexes found in the cytoplasm of microglia. Remarkably, nCUR therapy normalized the concentration of NF-kB, NLRP3, IL-1β, and IL-18 and significantly reduced the percentages of microglia displaying NLRP3 inflammasomes. The results implied that nCUR therapy inhibited inflammasome formation by modulating NF-kB activation, as CUR’s ability to modulate NF-kB has been observed in previous studies [88]. Such modulation likely also underlies the reduced concentration of multiple other proinflammatory cytokines and chemokines and diminished astrocyte hypertrophy and activated microglia observed in GWI rats receiving nCUR as NF-kB modulation can reduce proinflammatory markers, astrocyte hypertrophy and activated microglia [88-90].

The hippocampal neurogenesis was significantly reduced in GWI rats but enhanced with nCUR therapy with early and delayed nCUR treatment paradigms. Reduced neurogenesis at both early and extended time points after exposure to GWI-related chemicals and stress is consistent with our earlier reports [15-16, 24-25, 91]. The mechanisms underlying increased neurogenesis likely comprise both direct effects of nCUR on neural stem cell (NSC) proliferation and the indirect effects of nCUR by suppressing OS and neuroinflammation. nCUR has been shown to directly enhance the proliferation and differentiation of NSCs in the hippocampus through activation of the Wnt/β-catenin pathway [32]. Since neurogenesis is also sensitive to ROS levels and inflammatory milieu [24-25, 92], it is plausible that nCUR therapy promoted neurogenesis by improving the NSC milieu through suppression of ROS and neuroinflammation.

A moderate loss of the pre-synaptic protein Syn and increased expression of multiple genes involved in altered cognitive function were also seen in the hippocampus of rats with chronic GWI, similar to that observed in conditions such as dementia or Alzheimer’s disease (AD). These genes include *Bdnf, Fgf, Glun2B, Mgul5, Mapk1, Mapk3, Narg2, Npac, Pde4B, and CamKII*. The upregulation of Bdnf gene expression is surprising, considering several studies reporting reduced BDNF levels in GWI models [93-97]. However, it is not uncommon to have a discrepancy between mRNA and protein expressions. For example, significant age-related decreases in the concentration of FGF-2, BDNF, and VEGF proteins have been reported in the hippocampus with no changes in the expression *Fgf-2, Bdnf*, and *Vegf* genes [50, 98-100]. Increased Bdnf gene expression in GWI might be a compensatory reaction to the reduced concentration of BDNF. Alternatively, it could be due to altered posttranscriptional regulation mechanisms such as mRNA processing, export, localization, and translation [101], and/or protein modifications or degradation by altered levels of specific miRNAs [102]. Thus, additional studies on alterations in various stages of BDNF synthesis, including altered miRNA levels affecting precursor Bdnf (pro-BDNF) synthesis, and the rate of conversion of pro-BDNF into mature BDNF [103], will be needed to ascertain the causes of BDNF deficiency in chronic GWI.

The upregulation of *Fgf* could be a reaction to chronic neuroinflammation, as the protein that encodes the *Fgf* gene (FGFβ) is an antiinflammatory factor [104]. *Glun2B* and *mGLU5* are typically upregulated in neurodegenerative diseases, and likely contribute to cognitive dysfunction and excitotoxicity through enhanced glutamatergic neurotransmission [105-106]. Elevated *Mapk1* and *Mapk3* levels are associated with increased phosphorylation of tau, promoting neurodegeneration [107-109]. Increased *Narg2* expression denotes enhanced NMDA receptor activation or excitotoxicity, whereas elevated *Npac* increases p38 MAP kinase activity [110] involved in tau phosphorylation, neuroinflammation, and synaptic dysfunction [111]. Furthermore, *Pde4B* upregulation is associated with cognitive dysfunction in the early stage of AD [112], and increased *CamK2II* expression promotes neurodegeneration via tau [113]. Remarkably, twelve-weeks of nCUR treatment to rats with chronic GWI normalized the expression of all of the above genes and Syn in this study, implying the proficiency of nCUR to promote pro-cognitive effects.

Thus, multiple factors discussed above have likely contributed to persistent cognitive and mood dysfunction in rats with chronic GWI. Such a conclusion is supported by findings that impaired cognitive and mood dysfunction could occur from several molecular changes found in chronic GWI. These include cognitive and/or mood dysfunction observed after mitochondrial dysfunction [114], chronic neuroinflammation [27,115], increased levels of NLRP3 inflammasomes [116], reduced neurogenesis [117-118], and elevated expression of genes *Glun2B, Mgul5, Mapk1, Mapk3, Narg, Npac, Pde4B and CamKII*, as described above.

## Conclusions

The study demonstrated that low-dose (10 mg/Kg), intermittent, nCUR therapy commencing two months or eight months after exposure to GWI-related chemicals and stress is efficacious for improving cognitive and mood function. Remarkably, such recuperation was concomitant with substantial alleviation of OS, improved mitochondrial function, and dampened chronic neuroinflammation typified by NF-kB modulation and inhibition of NLPR3 inflammasomes. In addition, nCUR therapy enhanced neurogenesis, reduced synapse loss, and stabilized the expression of genes encoding proteins involved in cognitive dysfunction. These beneficial effects in an animal model of chronic GWI support the investigation of nCUR therapy for improving brain function in veterans with GWI through clinical trials. Due to its higher bioavailability than CUR after oral administration, nCUR therapy facilitates the use of a lower dose and reduced treatment frequency, which is ideal for reducing any potential toxic effects associated with long-term CUR treatment.

## References

[1] Dickey B, Madhu LN, Shetty AK (2021). Gulf War Illness: Mechanisms Underlying Brain Dysfunction and Promising Therapeutic Strategies. Pharmacol Ther, 220:107716. 33164782 10.1016/j.pharmthera.2020.107716 PMC8011829

[2] Steele L (2000). Prevalence and patterns of Gulf War illness in Kansas veterans: association of symptoms with characteristics of person, place, and time of military service. Am J Epidemiol, 152:992-1002. 11092441 10.1093/aje/152.10.992

[3] White RF, Steele L, O'Callaghan JP, Sullivan K, Binns JH, Golomb BA, et al. (2016). Recent research on Gulf War illness and other health problems in veterans of the 1991 Gulf War: Effects of toxicant exposures during deployment. Cortex, 74:449-475. 26493934 10.1016/j.cortex.2015.08.022 PMC4724528

[4] Committee on the Development of a Consensus Case Definition for Chronic Multisymptom Illness in - Gulf War V, Board on the Health of Select P, Institute of M. 2014. In Chronic Multisymptom Illness in Gulf War Veterans: Case Definitions Reexamined. Washington (DC).

[5] Fukuda K, Nisenbaum R, Stewart G, Thompson WW, Robin L, Washko RM, et al. (1998). Chronic multisymptom illness affecting Air Force veterans of the Gulf War. JAMA, 280:981-988. 9749480 10.1001/jama.280.11.981

[6] National Academies of Sciences E, Medicine, Health, Medicine D, Board on Population H, Public Health P, et al. (2018). In Gulf War and Health: Volume 11: Generational Health Effects of Serving in the Gulf War. Washington (DC). 30629394

[7] Bjørklund G, Pivina L, Dadar M, Semenova Y, Rahman MM, Chirumbolo S, et al. (2020). Depleted uranium and Gulf War Illness: Updates and comments on possible mechanisms behind the syndrome. Environ Res, 181:108927. 31796256 10.1016/j.envres.2019.108927

[8] Binns JH, Barlow C, Bloom FE, Clauw DJ, Golomb BA, Graves JC, et al. (2008). (Research Advisory Committee on Gulf War Veterans’ Illnesses) Gulf War Illness and the health of Gulf War Veterans Department of Veterans Affairs, Washington, (DC).

[9] Golomb BA (2008). Acetylcholinesterase inhibitors and Gulf War illnesses. Proc Natl Acad Sci U S A, 105:4295-4300. 18332428 10.1073/pnas.0711986105 PMC2393741

[10] Abdel-Rahman A, Abou-Donia S, El-Masry E, Shetty A, Abou-Donia M (2004). Stress and combined exposure to low doses of pyridostigmine bromide, DEET, and permethrin produce neurochemical and neuropathological alterations in cerebral cortex, hippocampus, and cerebellum. J Toxicol Environ Health A, 67:163-192. 14675905 10.1080/15287390490264802

[11] Abdel-Rahman A, Shetty AK, Abou-Donia MB (2001). Subchronic dermal application of N,N-diethyl m-toluamide (DEET) and permethrin to adult rats, alone or in combination, causes diffuse neuronal cell death and cytoskeletal abnormalities in the cerebral cortex and the hippocampus, and Purkinje neuron loss in the cerebellum. Exp Neurol, 172:153-171. 11681848 10.1006/exnr.2001.7807

[12] Abdel-Rahman A, Shetty AK, Abou-Donia MB (2002). Disruption of the blood-brain barrier and neuronal cell death in cingulate cortex, dentate gyrus, thalamus, and hypothalamus in a rat model of Gulf-War syndrome. Neurobiol Dis, 10:306-326. 12270692 10.1006/nbdi.2002.0524

[13] Alhasson F, Das S, Seth R, Dattaroy D, Chandrashekaran V, Ryan CN, et al. (2017). Altered gut microbiome in a mouse model of Gulf War Illness causes neuroinflammation and intestinal injury via leaky gut and TLR4 activation. PLoS One, 12:e0172914. 28328972 10.1371/journal.pone.0172914 PMC5362211

[14] Hattiangady B, Mishra V, Kodali M, Shuai B, Rao X, Shetty AK (2014). Object location and object recognition memory impairments, motivation deficits and depression in a model of Gulf War illness. Front Behav Neurosci, 8:78. 24659961 10.3389/fnbeh.2014.00078 PMC3952084

[15] Kodali M, Hattiangady B, Shetty GA, Bates A, Shuai B, Shetty AK (2018). Curcumin treatment leads to better cognitive and mood function in a model of Gulf War Illness with enhanced neurogenesis, and alleviation of inflammation and mitochondrial dysfunction in the hippocampus. Brain Behav Immun, 69:499-514. 29454881 10.1016/j.bbi.2018.01.009 PMC7023905

[16] Parihar VK, Hattiangady B, Shuai B, Shetty AK (2013). Mood and memory deficits in a model of Gulf War illness are linked with reduced neurogenesis, partial neuron loss, and mild inflammation in the hippocampus. Neuropsychopharmacology, 38:2348-2362. 23807240 10.1038/npp.2013.158 PMC3799073

[17] Shetty GA, Hattiangady B, Upadhya D, Bates A, Attaluri S, Shuai B, et al. (2017). Chronic Oxidative Stress, Mitochondrial Dysfunction, Nrf2 Activation and Inflammation in the Hippocampus Accompany Heightened Systemic Inflammation and Oxidative Stress in an Animal Model of Gulf War Illness. Front Mol Neurosci, 10:182. 28659758 10.3389/fnmol.2017.00182 PMC5469946

[18] Zakirova Z, Tweed M, Crynen G, Reed J, Abdullah L, Nissanka N, et al. (2015). Gulf War agent exposure causes impairment of long-term memory formation and neuropathological changes in a mouse model of Gulf War Illness. PLoS One, 10:e0119579. 25785457 10.1371/journal.pone.0119579 PMC4364893

[19] Abdullah L, Crynen G, Reed J, Bishop A, Phillips J, Ferguson S, et al. (2011). Proteomic CNS profile of delayed cognitive impairment in mice exposed to Gulf War agents. Neuromolecular Med, 13:275-288. 21986894 10.1007/s12017-011-8160-z

[20] Locker AR, Michalovicz LT, Kelly KA, Miller JV, Miller DB, O'Callaghan JP (2017). Corticosterone primes the neuroinflammatory response to Gulf War Illness-relevant organophosphates independently of acetylcholinesterase inhibition. J Neurochem, 142:444-455. 28500787 10.1111/jnc.14071 PMC5575502

[21] O'Callaghan JP, Kelly KA, Locker AR, Miller DB, Lasley SM (2015). Corticosterone primes the neuroinflammatory response to DFP in mice: potential animal model of Gulf War Illness. J Neurochem, 133:708-721. 25753028 10.1111/jnc.13088 PMC4722811

[22] Phillips KF, Deshpande LS (2016). Repeated low-dose organophosphate DFP exposure leads to the development of depression and cognitive impairment in a rat model of Gulf War Illness. Neurotoxicology, 52:127-133. 26619911 10.1016/j.neuro.2015.11.014

[23] Madhu LN, Attaluri S, Kodali M, Shuai B, Upadhya R, Gitai D, et al. (2019). Neuroinflammation in Gulf War Illness is linked with HMGB1 and complement activation, which can be discerned from brain-derived extracellular vesicles in the blood. Brain Behav Immun, 81:430-443. 31255677 10.1016/j.bbi.2019.06.040

[24] Shetty AK, Attaluri S, Kodali M, Shuai B, Shetty GA, Upadhya D, et al. (2020). Monosodium luminol reinstates redox homeostasis, improves cognition, mood and neurogenesis, and alleviates neuro- and systemic inflammation in a model of Gulf War Illness. Redox Biol, 28:101389. 31778892 10.1016/j.redox.2019.101389 PMC6888767

[25] Madhu LN, Kodali M, Attaluri S, Shuai B, Melissari L, Rao X, et al. (2021). Melatonin improves brain function in a model of chronic Gulf War Illness with modulation of oxidative stress, NLRP3 inflammasomes, and BDNF-ERK-CREB pathway in the hippocampus. Redox Biol, 43:101973. 33933884 10.1016/j.redox.2021.101973 PMC8105671

[26] Jenrow KA, Brown SL, Lapanowski K, Naei H, Kolozsvary A, Kim JH (2013). Selective inhibition of microglia-mediated neuroinflammation mitigates radiation-induced cognitive impairment. Radiat Res, 179:549-556. 23560629 10.1667/RR3026.1 PMC3673739

[27] Kohman RA, Rhodes JS (2013). Neurogenesis, inflammation and behavior. Brain Behav Immun, 27:22-32. 22985767 10.1016/j.bbi.2012.09.003 PMC3518576

[28] Tönnies E, Trushina E (2017). Oxidative Stress, Synaptic Dysfunction, and Alzheimer's Disease. J Alzheimers Dis, 57:1105-1121. 28059794 10.3233/JAD-161088 PMC5409043

[29] Hwang JY, Zukin RS (2018). REST, a master transcriptional regulator in neurodegenerative disease. Curr Opin Neurobiol, 48:193-200. 29351877 10.1016/j.conb.2017.12.008 PMC5892838

[30] Lu T, Pan Y, Kao SY, Li C, Kohane I, Chan J, et al. (2004). Gene regulation and DNA damage in the ageing human brain. Nature, 429:883-891. 15190254 10.1038/nature02661

[31] Kim SJ, Son TG, Park HR, Park M, Kim MS, Kim HS, et al. (2008). Curcumin stimulates proliferation of embryonic neural progenitor cells and neurogenesis in the adult hippocampus. J Biol Chem, 283:14497-14505. 18362141 10.1074/jbc.M708373200 PMC2386914

[32] Tiwari SK, Agarwal S, Seth B, Yadav A, Nair S, Bhatnagar P, et al. (2014). Curcumin-loaded nanoparticles potently induce adult neurogenesis and reverse cognitive deficits in Alzheimer's disease model via canonical Wnt/β-catenin pathway. ACS Nano, 8:76-103. 24467380 10.1021/nn405077y

[33] Xie Y, Zhao QY, Li HY, Zhou X, Liu Y, Zhang H (2014). Curcumin ameliorates cognitive deficits heavy ion irradiation-induced learning and memory deficits through enhancing of Nrf2 antioxidant signaling pathways. Pharmacol Biochem Behav, 126:181-186. 25159739 10.1016/j.pbb.2014.08.005

[34] Li Y, Li J, Li S, Li Y, Wang X, Liu B, et al. (2015). Curcumin attenuates glutamate neurotoxicity in the hippocampus by suppression of ER stress-associated TXNIP/NLRP3 inflammasome activation in a manner dependent on AMPK. Toxicol Appl Pharmacol, 286:53-63. 25791922 10.1016/j.taap.2015.03.010

[35] Ullah F, Liang A, Rangel A, Gyengesi E, Niedermayer G, Münch G (2017). High bioavailability curcumin: an anti-inflammatory and neurosupportive bioactive nutrient for neurodegenerative diseases characterized by chronic neuroinflammation. Arch Toxicol, 91:1623-1634. 28204864 10.1007/s00204-017-1939-4

[36] Anwar M, Ahmad I, Warsi MH, Mohapatra S, Ahmad N, Akhter S, et al. (2015). Experimental investigation and oral bioavailability enhancement of nano-sized curcumin by using supercritical anti-solvent process. Eur J Pharm Biopharm, 96:162-172. 26241925 10.1016/j.ejpb.2015.07.021

[37] Young NA, Bruss MS, Gardner M, Willis WL, Mo X, Valiente GR, et al. (2014). Oral administration of nano-emulsion curcumin in mice suppresses inflammatory-induced NFκB signaling and macrophage migration. PLoS One, 9:e111559. 25369140 10.1371/journal.pone.0111559 PMC4219720

[38] Ganugula R, Arora M, Jaisamut P, Wiwattanapatapee R, Jørgensen HG, Venkatpurwar VP, et al. (2017). Nano-curcumin safely prevents streptozotocin-induced inflammation and apoptosis in pancreatic beta cells for effective management of Type 1 diabetes mellitus. Br J Pharmacol, 174:2074-2084. 28409821 10.1111/bph.13816 PMC5466524

[39] Parihar VK, Hattiangady B, Kuruba R, Shuai B, Shetty AK (2011). Predictable chronic mild stress improves mood, hippocampal neurogenesis and memory. Mol Psychiatry, 16:171-183. 20010892 10.1038/mp.2009.130 PMC2891880

[40] Devadasu VR, Wadsworth RM, Kumar MN (2011). Protective effects of nanoparticulate coenzyme Q10 and curcumin on inflammatory markers and lipid metabolism in streptozotocin-induced diabetic rats: a possible remedy to diabetic complications. Drug Deliv Transl Res, 1:448-455. 25786365 10.1007/s13346-011-0041-3

[41] Grama CN, Suryanarayana P, Patil MA, Raghu G, Balakrishna N, Kumar MN, et al. (2013). Efficacy of biodegradable curcumin nanoparticles in delaying cataract in diabetic rat model. PLoS One, 8:e78217. 24155984 10.1371/journal.pone.0078217 PMC3796473

[42] Grama CN, Venkatpurwar VP, Lamprou DA, Ravi Kumar MN (2013). Towards scale-up and regulatory shelf-stability testing of curcumin encapsulated polyester nanoparticles. Drug Deliv Transl Res, 3:286-293. 25788136 10.1007/s13346-013-0150-2

[43] Hariharan S, Bhardwaj V, Bala I, Sitterberg J, Bakowsky U, Ravi Kumar MN (2006). Design of estradiol loaded PLGA nanoparticulate formulations: a potential oral delivery system for hormone therapy. Pharm Res, 23:184-195. 16267632 10.1007/s11095-005-8418-y

[44] Shaikh J, Ankola DD, Beniwal V, Singh D, Kumar MN (2009). Nanoparticle encapsulation improves oral bioavailability of curcumin by at least 9-fold when compared to curcumin administered with piperine as absorption enhancer. Eur J Pharm Sci, 37:223-230. 19491009 10.1016/j.ejps.2009.02.019

[45] Sengupta P (2013). The Laboratory Rat: Relating Its Age with Human's. Int J Prev Med, 4:624-630. 23930179 PMC3733029

[46] Hattiangady B, Kuruba R, Shetty AK (2011). Acute Seizures in Old Age Leads to a Greater Loss of CA1 Pyramidal Neurons, an Increased Propensity for Developing Chronic TLE and a Severe Cognitive Dysfunction. Aging Dis, 2:1-17. 21339903 PMC3041587

[47] Kodali M, Parihar VK, Hattiangady B, Mishra V, Shuai B, Shetty AK (2015). Resveratrol prevents age-related memory and mood dysfunction with increased hippocampal neurogenesis and microvasculature, and reduced glial activation. Sci Rep, 5:8075. 25627672 10.1038/srep08075 PMC4894403

[48] Rao MS, Hattiangady B, Shetty AK (2008). Status epilepticus during old age is not associated with enhanced hippocampal neurogenesis. Hippocampus, 18:931-944. 18493929 10.1002/hipo.20449 PMC3612499

[49] Mishra V, Shuai B, Kodali M, Shetty GA, Hattiangady B, Rao X, et al. (2015). Resveratrol Treatment after Status Epilepticus Restrains Neurodegeneration and Abnormal Neurogenesis with Suppression of Oxidative Stress and Inflammation. Sci Rep, 5:17807. 26639668 10.1038/srep17807 PMC4671086

[50] Shetty GA, Hattiangady B, Shetty AK (2013). Neural stem cell- and neurogenesis-related gene expression profiles in the young and aged dentate gyrus. Age (Dordr), 35:2165-2176. 23322452 10.1007/s11357-012-9507-6 PMC3824978

[51] Upadhya R, Madhu LN, Attaluri S, Gitaí DLG, Pinson MR, Kodali M, et al. (2020). Extracellular vesicles from human iPSC-derived neural stem cells: miRNA and protein signatures, and anti-inflammatory and neurogenic properties. J Extracell Vesicles, 9:1809064. 32944193 10.1080/20013078.2020.1809064 PMC7480597

[52] Kodali M, Megahed T, Mishra V, Shuai B, Hattiangady B, Shetty AK (2016). Voluntary Running Exercise-Mediated Enhanced Neurogenesis Does Not Obliterate Retrograde Spatial Memory. J Neurosci, 36:8112-8122. 27488632 10.1523/JNEUROSCI.0766-16.2016 PMC6601951

[53] Shetty AK, Hattiangady B, Rao MS, Shuai B (2012). Neurogenesis response of middle-aged hippocampus to acute seizure activity. PLoS One, 7:e43286. 22912847 10.1371/journal.pone.0043286 PMC3422269

[54] Hattiangady B, Shuai B, Cai J, Coksaygan T, Rao MS, Shetty AK (2007). Increased dentate neurogenesis after grafting of glial restricted progenitors or neural stem cells in the aging hippocampus. Stem Cells, 25:2104-2117. 17510219 10.1634/stemcells.2006-0726

[55] Rao MS, Shetty AK (2004). Efficacy of doublecortin as a marker to analyze the absolute number and dendritic growth of newly generated neurons in the adult dentate gyrus. Eur J Neurosci, 19:234-246. 14725617 10.1111/j.0953-816x.2003.03123.x

[56] Kodali M, Attaluri S, Madhu LN, Shuai B, Upadhya R, Gonzalez JJ, et al. (2021). Metformin treatment in late middle age improves cognitive function with alleviation of microglial activation and enhancement of autophagy in the hippocampus. Aging Cell, 20:e13277. 33443781 10.1111/acel.13277 PMC7884047

[57] Engdahl BE, James LM, Miller RD, Leuthold AC, Lewis SM, Carpenter AF, et al. (2018). Brain Function in Gulf War Illness (GWI) and Associated Mental Health Comorbidities. J Neurol Neuromedicine, 3:24-34. 30882065 PMC6417922

[58] Janulewicz PA, Krengel MH, Maule A, White RF, Cirillo J, Sisson E, et al. (2017). Neuropsychological characteristics of Gulf War illness: A meta-analysis. PLoS One, 12:e0177121. 28520755 10.1371/journal.pone.0177121 PMC5435307

[59] Odegard TN, Cooper CM, Farris EA, Arduengo J, Bartlett J, Haley R (2013). Memory impairment exhibited by veterans with Gulf War Illness. Neurocase, 19:316-327. 22519425 10.1080/13554794.2012.667126

[60] Gopinath KS, Sakoglu U, Crosson BA, Haley RW (2019). Exploring brain mechanisms underlying Gulf War Illness with group ICA based analysis of fMRI resting state networks. Neurosci Lett, 701:136-141. 30825590 10.1016/j.neulet.2019.02.041

[61] Joshi U, Pearson A, Evans JE, Langlois H, Saltiel N, Ojo J, et al. (2019). A permethrin metabolite is associated with adaptive immune responses in Gulf War Illness. Brain Behav Immun, 81:545-559. 31325531 10.1016/j.bbi.2019.07.015 PMC7155744

[62] Michalovicz LT, Locker AR, Kelly KA, Miller JV, Barnes Z, Fletcher MA, et al. (2019). Corticosterone and pyridostigmine/DEET exposure attenuate peripheral cytokine expression: Supporting a dominant role for neuroinflammation in a mouse model of Gulf War Illness. Neurotoxicology, 70:26-32. 30339781 10.1016/j.neuro.2018.10.006 PMC6533534

[63] O'Callaghan JP, Michalovicz LT, Kelly KA (2016). Supporting a Neuroimmune Basis of Gulf War Illness. EBioMedicine, 13:5-6. 27806904 10.1016/j.ebiom.2016.10.037 PMC5264477

[64] Parkitny L, Middleton S, Baker K, Younger J (2015). Evidence for abnormal cytokine expression in Gulf War Illness: A preliminary analysis of daily immune monitoring data. BMC Immunol, 16:57. 26420016 10.1186/s12865-015-0122-z PMC4589096

[65] Baraniuk JN, El-Amin S, Corey R, Rayhan R, Timbol C (2013). Carnosine treatment for gulf war illness: a randomized controlled trial. Glob J Health Sci, 5:69-81. 23618477 10.5539/gjhs.v5n3p69 PMC4209301

[66] Koslik HJ, Hamilton G, Golomb BA (2014). Mitochondrial dysfunction in Gulf War illness revealed by 31Phosphorus Magnetic Resonance Spectroscopy: a case-control study. PLoS One, 9:e92887. 24675771 10.1371/journal.pone.0092887 PMC3968048

[67] Abou-Donia MB, Conboy LA, Kokkotou E, Jacobson E, Elmasry EM, Elkafrawy P, et al. (2017). Screening for novel central nervous system biomarkers in veterans with Gulf War Illness. Neurotoxicol Teratol, 61:36-46. 28286177 10.1016/j.ntt.2017.03.002

[68] Alshelh Z, Albrecht DS, Bergan C, Akeju O, Clauw DJ, Conboy L, et al. (2020). In-vivo imaging of neuroinflammation in veterans with Gulf War illness. Brain Behav Immun, 87:498-507. 32027960 10.1016/j.bbi.2020.01.020 PMC7864588

[69] Johnson GJ, Slater BC, Leis LA, Rector TS, Bach RR (2016). Blood Biomarkers of Chronic Inflammation in Gulf War Illness. PLoS One, 11:e0157855. 27352030 10.1371/journal.pone.0157855 PMC4924830

[70] Tsikas D (2017). Assessment of lipid peroxidation by measuring malondialdehyde (MDA) and relatives in biological samples: Analytical and biological challenges. Anal Biochem, 524:13-30. 27789233 10.1016/j.ab.2016.10.021

[71] Weber D, Davies MJ, Grune T (2015). Determination of protein carbonyls in plasma, cell extracts, tissue homogenates, isolated proteins: Focus on sample preparation and derivatization conditions. Redox Biol, 5:367-380. 26141921 10.1016/j.redox.2015.06.005 PMC4506980

[72] Joshi G, Johnson JA (2012). The Nrf2-ARE pathway: a valuable therapeutic target for the treatment of neurodegenerative diseases. Recent Pat CNS Drug Discov, 7:218-229. 22742419 10.2174/157488912803252023 PMC3625035

[73] Rouzier C, Chaussenot A, Fragaki K, Serre V, Ait-El-Mkadem S, Richelme C, et al. (2019). NDUFS6 related Leigh syndrome: a case report and review of the literature. J Hum Genet, 64:637-645. 30948790 10.1038/s10038-019-0594-4

[74] Hinson JT, Fantin VR, Schönberger J, Breivik N, Siem G, McDonough B, et al. (2007). Missense mutations in the BCS1L gene as a cause of the Björnstad syndrome. N Engl J Med, 356:809-819. 17314340 10.1056/NEJMoa055262

[75] Sato A, Takagi K, Miki Y, Yoshimura A, Hara M, Ishida T, et al. (2019). Cytochrome c1 as a favorable prognostic marker in estrogen receptor-positive breast carcinoma. Histol Histopathol, 34:1365-1375. 31149728 10.14670/HH-18-130

[76] Lazarou M, Smith SM, Thorburn DR, Ryan MT, McKenzie M (2009). Assembly of nuclear DNA-encoded subunits into mitochondrial complex IV, and their preferential integration into supercomplex forms in patient mitochondria. FEBS J, 276:6701-6713. 19843159 10.1111/j.1742-4658.2009.07384.x

[77] Tvina A, Thomsen A, Palatnik A (2020). Prenatal and postnatal phenotype of a pathologic variant in the ATP6AP1 gene. Eur J Med Genet, 63:103881. 32058063 10.1016/j.ejmg.2020.103881

[78] Guo C, Sun L, Chen X, Zhang D (2013). Oxidative stress, mitochondrial damage and neurodegenerative diseases. Neural Regen Res, 8:2003-2014. 25206509 10.3969/j.issn.1673-5374.2013.21.009 PMC4145906

[79] Samarghandian S, Azimi-Nezhad M, Farkhondeh T, Samini F (2017). Anti-oxidative effects of curcumin on immobilization-induced oxidative stress in rat brain, liver and kidney. Biomed Pharmacother, 87:223-229. 28061405 10.1016/j.biopha.2016.12.105

[80] Akinyemi AJ, Oboh G, Ogunsuyi O, Abolaji AO, Udofia A (2018). Curcumin-supplemented diets improve antioxidant enzymes and alter acetylcholinesterase genes expression level in Drosophila melanogaster model. Metab Brain Dis, 33:369-375. 28849357 10.1007/s11011-017-0100-7

[81] Dong W, Yang B, Wang L, Li B, Guo X, Zhang M, et al. (2018). Curcumin plays neuroprotective roles against traumatic brain injury partly via Nrf2 signaling. Toxicol Appl Pharmacol, 346:28-36. 29571711 10.1016/j.taap.2018.03.020

[82] Hagl S, Kocher A, Schiborr C, Kolesova N, Frank J, Eckert GP (2015). Curcumin micelles improve mitochondrial function in neuronal PC12 cells and brains of NMRI mice - Impact on bioavailability. Neurochem Int, 89:234-242. 26254982 10.1016/j.neuint.2015.07.026

[83] Broz P, Dixit VM (2016). Inflammasomes: mechanism of assembly, regulation and signalling. Nat Rev Immunol, 16:407-420. 27291964 10.1038/nri.2016.58

[84] Voet S, Srinivasan S, Lamkanfi M, van Loo G (2019). Inflammasomes in neuroinflammatory and neurodegenerative diseases. EMBO Mol Med, 11, e10248. 31015277 10.15252/emmm.201810248 PMC6554670

[85] Canesin G, Hejazi SM, Swanson KD, Wegiel B (2020). Heme-Derived Metabolic Signals Dictate Immune Responses. Front Immunol, 11:66. 32082323 10.3389/fimmu.2020.00066 PMC7005208

[86] Lamkanfi M, Dixit VM (2012). Inflammasomes and their roles in health and disease. Annu Rev Cell Dev Biol, 28:137-161. 22974247 10.1146/annurev-cellbio-101011-155745

[87] Yang J, Liu Z, Xiao TS (2017). Post-translational regulation of inflammasomes. Cell Mol Immunol, 14:65-79. 27345727 10.1038/cmi.2016.29 PMC5214939

[88] Zhang J, Zheng Y, Luo Y, Du Y, Zhang X, Fu J (2019). Curcumin inhibits LPS-induced neuroinflammation by promoting microglial M2 polarization via TREM2/TLR4/NF-κB pathways in BV2 cells. Mol Immunol, 116:29-37. 31590042 10.1016/j.molimm.2019.09.020

[89] Lawrence T (2009). The nuclear factor NF-kappaB pathway in inflammation. Cold Spring Harb Perspect Biol, 1:a001651. 20457564 10.1101/cshperspect.a001651 PMC2882124

[90] Saggu R, Schumacher T, Gerich F, Rakers C, Tai K, Delekate A, et al. (2016). Astroglial NF-kB contributes to white matter damage and cognitive impairment in a mouse model of vascular dementia. Acta Neuropathol Commun, 4:76. 27487766 10.1186/s40478-016-0350-3 PMC4973061

[91] Megahed T, Hattiangady B, Shuai B, Shetty AK (2014). Parvalbumin and neuropeptide Y expressing hippocampal GABA-ergic inhibitory interneuron numbers decline in a model of Gulf War illness. Front Cell Neurosci, 8:447. 25620912 10.3389/fncel.2014.00447 PMC4288040

[92] Huang TT, Leu D, Zou Y (2015). Oxidative stress and redox regulation on hippocampal-dependent cognitive functions. Arch Biochem Biophys, 576:2-7. 25797440 10.1016/j.abb.2015.03.014 PMC4456256

[93] Bose D, Saha P, Mondal A, Fanelli B, Seth RK, Janulewicz P, et al. (2020). Obesity Worsens Gulf War Illness Symptom Persistence Pathology by Linking Altered Gut Microbiome Species to Long-Term Gastrointestinal, Hepatic, and Neuronal Inflammation in a Mouse Model. Nutrients, 12, 2764. 10.3390/nu12092764 PMC7551189 32927823

[94] Carreras I, Aytan N, Mellott T, Choi JK, Lehar M, Crabtree L, et al. (2018). Anxiety, neuroinflammation, cholinergic and GABAergic abnormalities are early markers of Gulf War illness in a mouse model of the disease. Brain Res, 1681:34-43. 29277710 10.1016/j.brainres.2017.12.030 PMC5971846

[95] Kimono D, Bose D, Seth RK, Mondal A, Saha P, Janulewicz P, et al. (2020). Host Akkermansia muciniphila Abundance Correlates With Gulf War Illness Symptom Persistence via NLRP3-Mediated Neuroinflammation and Decreased Brain-Derived Neurotrophic Factor. Neurosci Insights, 15:2633105520942480. 32832901 10.1177/2633105520942480 PMC7440889

[96] Ribeiro ACR, Zhu J, Kronfol MM, Jahr FM, Younis RM, Hawkins E, et al. (2020). Molecular mechanisms for the antidepressant-like effects of a low-dose ketamine treatment in a DFP-based rat model for Gulf War Illness. Neurotoxicology, 80:52-59. 32592718 10.1016/j.neuro.2020.06.011

[97] Ribeiro ACR, Jahr FM, Hawkins E, Kronfol MM, Younis RM, McClay JL, et al. (2021). Epigenetic histone acetylation and Bdnf dysregulation in the hippocampus of rats exposed to repeated, low-dose diisopropylfluorophosphate. Life Sci, 281:119765. 34186043 10.1016/j.lfs.2021.119765

[98] Hattiangady B, Rao MS, Shetty GA, Shetty AK (2005). Brain-derived neurotrophic factor, phosphorylated cyclic AMP response element binding protein and neuropeptide Y decline as early as middle age in the dentate gyrus and CA1 and CA3 subfields of the hippocampus. Exp Neurol, 195:353-371. 16002067 10.1016/j.expneurol.2005.05.014

[99] Shetty AK, Hattiangady B, Shetty GA (2005). Stem/progenitor cell proliferation factors FGF-2, IGF-1, and VEGF exhibit early decline during the course of aging in the hippocampus: role of astrocytes. Glia, 51:173-186. 15800930 10.1002/glia.20187

[100] Bernal GM, Peterson DA (2011). Phenotypic and gene expression modification with normal brain aging in GFAP-positive astrocytes and neural stem cells. Aging Cell, 10:466-482. 21385309 10.1111/j.1474-9726.2011.00694.x PMC3094510

[101] Gingold H, Pilpel Y (2011). Determinants of translation efficiency and accuracy. Mol Syst Biol, 7:481. 21487400 10.1038/msb.2011.14 PMC3101949

[102] Schouten M, Buijink MR, Lucassen PJ, Fitzsimons CP (2012). New Neurons in Aging Brains: Molecular Control by Small Non-Coding RNAs. Front Neurosci, 6:25. 22363255 10.3389/fnins.2012.00025 PMC3281214

[103] Greenberg ME, Xu B, Lu B, Hempstead BL (2009). New insights in the biology of BDNF synthesis and release: implications in CNS function. J Neurosci, 29:12764-12767. 19828787 10.1523/JNEUROSCI.3566-09.2009 PMC3091387

[104] Tang MM, Lin WJ, Pan YQ, Li YC (2018). Fibroblast Growth Factor 2 Modulates Hippocampal Microglia Activation in a Neuroinflammation Induced Model of Depression. Front Cell Neurosci, 12:255. 30135647 10.3389/fncel.2018.00255 PMC6092504

[105] Hamilton A, Vasefi M, Vander Tuin C, McQuaid RJ, Anisman H, Ferguson SS (2016). Chronic Pharmacological mGluR5 Inhibition Prevents Cognitive Impairment and Reduces Pathogenesis in an Alzheimer Disease Mouse Model. Cell Rep, 15:1859-1865. 27210751 10.1016/j.celrep.2016.04.077

[106] Raybuck JD, Hargus NJ, Thayer SA (2017). A GluN2B-Selective NMDAR Antagonist Reverses Synapse Loss and Cognitive Impairment Produced by the HIV-1 Protein Tat. J Neurosci, 37:7837-7847. 28716964 10.1523/JNEUROSCI.0226-17.2017 PMC5559761

[107] Franco R, Martínez-Pinilla E, Navarro G, Zamarbide M (2017). Potential of GPCRs to modulate MAPK and mTOR pathways in Alzheimer's disease. Prog Neurobiol, 149-150:21-38. 28189739 10.1016/j.pneurobio.2017.01.004

[108] Gerschütz A, Heinsen H, Grünblatt E, Wagner AK, Bartl J, Meissner C, et al. (2014). Neuron-specific alterations in signal transduction pathways associated with Alzheimer's disease. J Alzheimers Dis, 40:135-142. 24334724 10.3233/JAD-131280

[109] Kheiri G, Dolatshahi M, Rahmani F, Rezaei N (2018). Role of p38/MAPKs in Alzheimer's disease: implications for amyloid beta toxicity targeted therapy. Rev Neurosci, 30:9-30. 29804103 10.1515/revneuro-2018-0008

[110] Fu J, Yang Z, Wei J, Han J, Gu J (2006). Nuclear protein NP60 regulates p38 MAPK activity. J Cell Sci, 119:115-123. 16352664 10.1242/jcs.02699

[111] Lee JK, Kim NJ (2017). Recent Advances in the Inhibition of p38 MAPK as a Potential Strategy for the Treatment of Alzheimer's Disease. Molecules, 22, 1287. 10.3390/molecules22081287 PMC6152076 28767069

[112] Richter W, Menniti FS, Zhang HT, Conti M (2013). PDE4 as a target for cognition enhancement. Expert Opin Ther Targets, 17:1011-1027. 23883342 10.1517/14728222.2013.818656 PMC4066988

[113] Oka M, Fujisaki N, Maruko-Otake A, Ohtake Y, Shimizu S, Saito T, et al. (2017). Ca2+/calmodulin-dependent protein kinase II promotes neurodegeneration caused by tau phosphorylated at Ser262/356 in a transgenic Drosophila model of tauopathy. J Biochem, 162:335-342. 28992057 10.1093/jb/mvx038 PMC5892399

[114] Fernandez A, Meechan DW, Karpinski BA, Paronett EM, Bryan CA, Rutz HL, et al. (2019). Mitochondrial Dysfunction Leads to Cortical Under-Connectivity and Cognitive Impairment. Neuron, 102:1127-1142.e1123. 31079872 10.1016/j.neuron.2019.04.013 PMC6668992

[115] Kaltschmidt B, Kaltschmidt C (2015). NF-KappaB in Long-Term Memory and Structural Plasticity in the Adult Mammalian Brain. Front Mol Neurosci, 8:69. 26635522 10.3389/fnmol.2015.00069 PMC4656838

[116] Ward R, Li W, Abdul Y, Jackson L, Dong G, Jamil S, et al. (2019). NLRP3 inflammasome inhibition with MCC950 improves diabetes-mediated cognitive impairment and vasoneuronal remodeling after ischemia. Pharmacol Res, 142:237-250. 30818045 10.1016/j.phrs.2019.01.035 PMC6486792

[117] McAvoy KM, Sahay A (2017). Targeting Adult Neurogenesis to Optimize Hippocampal Circuits in Aging. Neurotherapeutics, 14:630-645. 28536851 10.1007/s13311-017-0539-6 PMC5509633

[118] Peng L, Bonaguidi MA (2018). Function and Dysfunction of Adult Hippocampal Neurogenesis in Regeneration and Disease. Am J Pathol, 188:23-28. 29030053 10.1016/j.ajpath.2017.09.004 PMC5745527

